# Lipid metabolism disorder in diabetic kidney disease

**DOI:** 10.3389/fendo.2024.1336402

**Published:** 2024-04-29

**Authors:** Yi-Zhen Han, Bo-Xuan Du, Xing-Yu Zhu, Yang-Zhi-Yuan Wang, Hui-Juan Zheng, Wei-Jing Liu

**Affiliations:** ^1^Dongzhimen Hospital, Beijing University of Chinese Medicine, Beijing, China; ^2^School of Acupuncture-Moxibustion and Tuina, Beijing University of Chinese Medicine, Beijing, China

**Keywords:** diabetic kidney disease, ferroptosis, gut microbiota, lipid metabolism, metabolic reprogramming, treatment

## Abstract

Diabetic kidney disease (DKD), a significant complication associated with diabetes mellitus, presents limited treatment options. The progression of DKD is marked by substantial lipid disturbances, including alterations in triglycerides, cholesterol, sphingolipids, phospholipids, lipid droplets, and bile acids (BAs). Altered lipid metabolism serves as a crucial pathogenic mechanism in DKD, potentially intertwined with cellular ferroptosis, lipophagy, lipid metabolism reprogramming, and immune modulation of gut microbiota (thus impacting the liver-kidney axis). The elucidation of these mechanisms opens new potential therapeutic pathways for DKD management. This research explores the link between lipid metabolism disruptions and DKD onset.

## 1 Introduction

Diabetic kidney disease (DKD) is a common microvascular complication of diabetes mellitus (1). DKD is characterized by glomerulopathy with diffuse and nodular tethered dilatation and thickening of the glomerular basement membranes, accompanied by tubular atrophy, interstitial inflammation, fibrosis, glomerular endothelial injury, podocyte loss, and glomerular vascular hyalinopathy (2). DKD pathogenesis is complex and is associated with glucose and lipid metabolism disorders and stress (3, 4). Treatments, such as glycemic control and urinary albumin reduction, do not fundamentally alter the course of DKD (5). The latest evidence-based guidelines recommend angiotensin-converting enzyme inhibitors (ACEIs)/angiotensin receptor blockers (ARBs) and novel hypoglycemic agents, such as dipeptidyl peptidase-4 inhibitors, sodium-glucose transporter 2 inhibitors, and sodium-glucose transporter 2 inhibitors. Sodium-glucose transporter 2 inhibitors and glucagon-like peptide 1 agonists (6–8) have not been found to slow down the progression of DKD to end-stage renal disease (1). Therefore, exploring the pathogenesis of DKD and identifying targets for intervention are important clinical goals. Lipid metabolism disorders, one of the pathogenic mechanisms of DKD, mainly involve abnormalities in lipid metabolism, such as triglycerides (TGs), cholesterol (CHOL), and lipid droplets (LDs) (9–11). Recently, there have been numerous studies on the mechanisms of lipid metabolism disorder lipophagy in DKD. Ferroptosis, the programmed death in DKD, is a direct result of lipid peroxidation, which is closely related to lipid metabolism disorders, especially disorders in the regulation of fatty acids (FAs) (12, 13). Another mode of programmed death is associated with defects in the autophagy-lysosomal system and abnormal lipid accumulation in podocytes in DKD (14, 15). The reprogramming of lipid metabolism also results in dysfunctional lipid uptake and oxidation, especially of FAs, which are exacerbated in DKD (16, 17). In addition, an imbalance of gut microbiota and increased permeability of the intestinal barrier, which is one of the pathological manifestations of DKD, and its involvement in immune imbalance, especially involving the liver-kidney axis, affect lipid metabolism, such as bile acid (BA) metabolism, and the immune imbalance aggravates renal injury (18–20). Recent studies related to lipid metabolism disorders in human and animal models of DKD have revealed that the deposition of toxic FAs metabolites leads to ectopic lipid deposition in podocytes and tubular epithelial cells, interstitial fibrosis, and DKD (17, 21–23). The causes and pathogenesis of lipid metabolism disorders in DKD have not yet been fully elucidated. Therefore, studying the mechanism of lipid metabolism changes in DKD and how to slow down the development of DKD through the regulation of different targets has become a hot research topic.

## 2 Characterization of lipid metabolism changes in DKD

Abnormalities in the metabolism of TG, CHOL, sphingolipids, phospholipids (PLs), LDs, and BAs are key factors in DKD progression. Both the quality and quantity of lipids are associated with this process and produce reactive oxygen species (ROS), which exacerbate oxidative stress, inflammation, and cell death (24).

### 2.1 Abnormal TG metabolism in DKD

Abnormal TG metabolism in DKD is mainly characterized by abnormal uptake and oxidation of FAs. Fatty acid transport proteins(FATPs),cluster of differentiation 36 (CD36), and fatty acid-binding protein (FABP) are correlated with FA uptake in DKD. FATPs control FAs uptake, and fatty acid transport protein 2 (FATP2) deficiency improves renal outcomes (25, 26). fatty acid transport protein 4(FATP4) levels in diabetic mice are correlated with lipid accumulation in DKD (27). CD36 is a transmembrane glycoprotein that mediates oxidized low-density lipoprotein (LDL) uptake. An increase in CD36 levels is strongly associated with kidney injury in DKD (28–32). Increased CD36 expression in mouse kidneys promotes TG accumulation in the kidney (33). fatty acid-binding protein1(FABP1), another protein associated with abnormal lipid uptake in DKD, is a reliable marker of the onset and progression of DKD (34–37).

Fatty acid oxidation (FAO) is the primary pathway that reduces the renal lipid content. The expression of FAO genes, including peroxisome proliferators-activated receptors α (PPARα), acyl coenzyme A dehydrogenase, and acyl-CoA oxidase 1/2(ACOX1/2), was significantly reduced (33, 38).

Non esterified fatty acids (NEFA) and essential fatty acid (EFA) changes are also important in DKD. In the early stages of DKD, NEFAs increase and EFAs decrease (39). Other NEFAs (Monounsaturated 16:1/18:1 FAs, omega-6/7/9 in the serum, and 10-nitrooleic acid in the urine) are also consistently elevated in DKD (40). In addition, long-chain free fatty acid levels were reduced in rats with DKD (41).

### 2.2 Abnormal CHOL metabolism in DKD

CHOL synthesis, endocytosis, and exocytosis are all closely associated with DKD. Studies have demonstrated that increased expression of sterol regulatory element-binding proteins (SREBP) and isoforms associated with CHOL synthesis that mediate intracellular CHOL sensing leads to renal damage in DKD (42–46) and plays a role in the accumulation of LDs (47). Increased expression of SREBP and its isoforms in glomeruli of patients with DKD leads to renal injury (48–50). Inhibition of CHOL efflux and increased CHOL influx in DKD cells increases free CHOL levels, which activate sterol O-acyltransferase 1 to form cholesteryl esters (ChEs) that are stored in LDs, causing excessive accumulation of CHOL in podocytes (22, 44, 51). In contrast, induction of CHOL efflux ameliorates DKD progression and DKD-like glomerulosclerosis (38, 52). ATP-binding cassette transporter A1 (ABCA1), which promotes CHOL efflux, and another CHOL efflux scavenger receptor, BI (SR-BI), were found to be significantly inhibited in DKD (53).

### 2.3 Sphingolipids anomalies in DKD

Expression of the sphingolipids metabolites ceramide (Cer), sphingosine-1-phosphate (S1P), Ceramide-1-phosphate (C1P) is specific to DKD. Long-chain Cer and ultra-long-chain Cer levels are elevated in DKD (54–56). Renal S1P levels are elevated in diabetic mice (57, 58), and sphingosine kinase, which produces S1P, exhibits increased expression and activity (59). Receptor signaling for S1P is specifically expressed during glomerular injury (60, 61). Sphingomyelin phosphodiesterase acid-like 3b (SMPDL3b) is increased in DKD mice in association with a C1P-deficient state in podocytes (62–64). In addition, increased ganglioside GM3(GM3) in the renal cortex during the early stages of diabetes alters pro-survival receptor-related Automatic Kernel Tunables (Akt) and Protein kinase B signaling to exacerbate DKD (65–68). This suggests a role for sphingolipids in the development of DKD.

### 2.4 Abnormal metabolism of PLs in DKD

PLs are key structural components of all cellular lipid bilayers that contain multiple fatty acyl groups and are potential biomarkers of DKD (69–72). Phosphatidylethanolamine (PE), phosphatidylinositol (PI), phosphocholine (PC) and sphingomyelin (SM) were significantly altered (73, 74). Levels of two lysolecithins (PC and lysophosphatidic acid (LPA)) and Sphingomyelin (SM) (d18:1/16:0) were found to be significantly elevated in the glomeruli of diabetic mice (75–77), and the levels of glucose-modified aminoketoses (Amadori-PEs) were even higher in the renal cortex. PI (40:6) levels tended to decrease in the serum of patients with type 2 DKD (78). In addition, it has been shown that diabetic mice also show reduced relative abundance of Cardiolipin (CL) and its subpopulations in the proximal tubules of the renal cortex (79).

### 2.5 Accumulation of LDs in DKD

LDs are cellular reservoirs of CHOL and acylglycerols (80). LDs alleviate DKD by preventing lipotoxicity and lipid apoptosis (81–83) or enhancing autophagic pathways (84). Increased accumulation of LDs in DKD was found (22, 52, 85),and an increase in LDs in glomerular and/or tubular cells of the kidneys of hyperglycemic mice was accompanied by an increase in markers of oxidative stress (xanthine oxidoreductase (XOR) and nitrotyrosine with tail-interacting protein of 47 kDa (TIP47)) (86). The expression of perilipin 2 (PLIN2), a family of lipoproteins present in the coating of LDs, is significantly upregulated in DKD pedunculated cells (27, 87).

### 2.6 Abnormal metabolism of BAs in DKD

**Figure 1:**
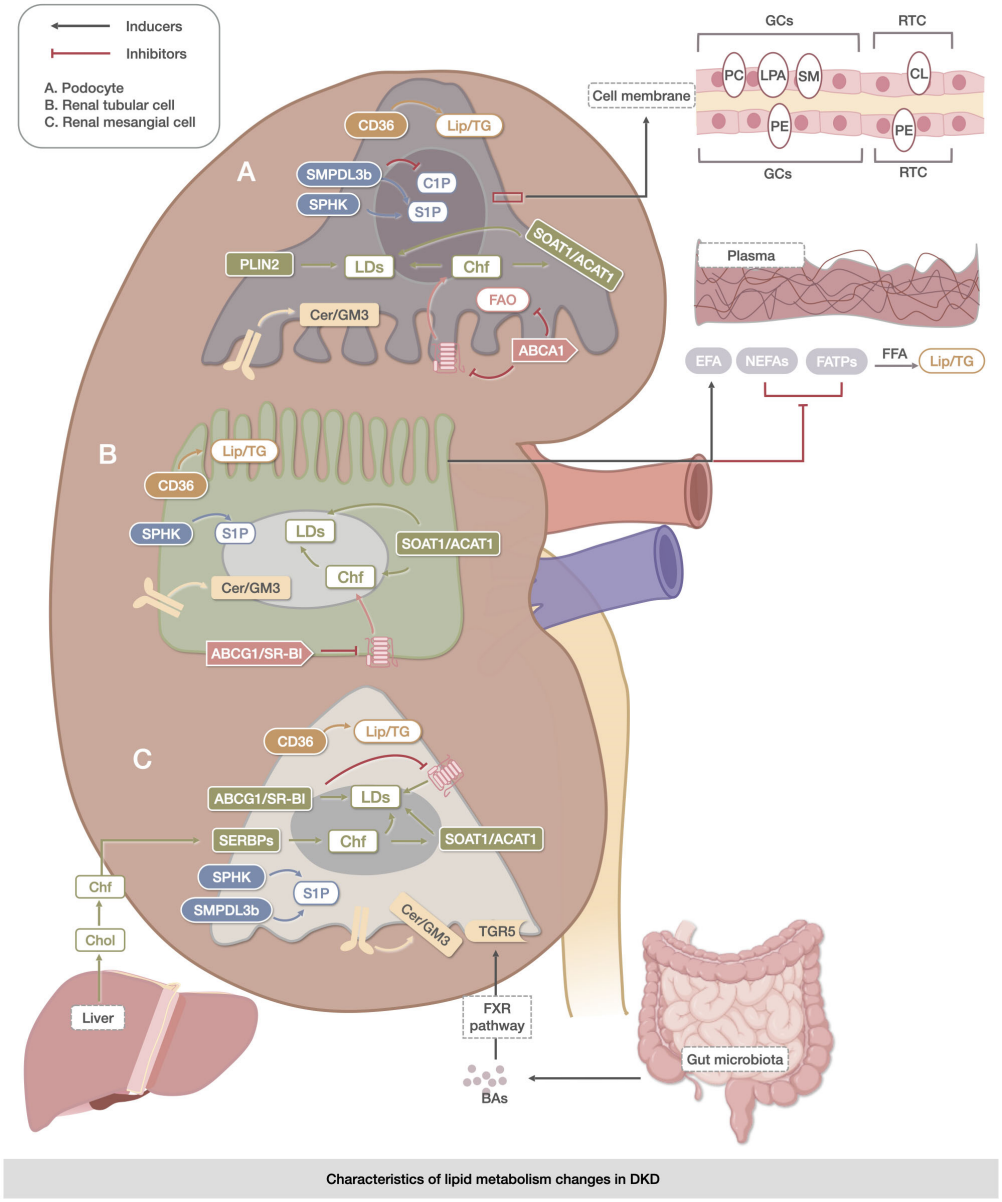
Characteristics of lipid metabolism changes in DKD. The metabolic abnormalities of TG, CHOL, sphingolipids, PLs, LDs, and BAs were mainly reflected in the metabolic abnormalities of TG, which were reflected in the uptake and oxidation process of FAs. The abnormalities of CHOL were related to its own synthesis, endocytosis, and exocytosis. The expression of the metabolites of sphingolipids, Cer, S1P, and C1P was specific to DKD. The metabolic abnormalities of PLs (PC, LPA, SM, CL, PE, and PI) were significantly altered in kidney-associated cell membranes. Changes in LDs were mainly associated with the accumulation of Lipid in DKD cells. BAs may delay renal injury through direct activation of the FXR pathway and TGR5 membrane receptors. The upregulation of CD36 expression facilitated triglyceride accumulation in the kidney, while the increase in SMPDL3b was linked to ceramide-1-phosphate deficiency in podocytes. The coordinated actions of SOAT1/ACAT1, ABCG1/SR-B1, and ABCG1/SR-B were involved in lipid droplet accumulation. However, dysregulation of Akt and protein kinase B signaling by Cer/GM3 exacerbated lipid metabolism abnormalities in renal podocytes, tubule cells, and mesangial cells. These processes are closely associated with the intestine, liver, and blood vessels.

BAs are oxidized hepatic enzymes derived from CHOL and are found mainly in the enterohepatic circulatory system; they may be directly involved in the regulation of blood glucose (88) or indirectly involved through the gut-kidney axis, improving lipid metabolism to protect the kidney (89). BAs and total CHOL were negatively correlated with the severity of DKD, and BAs may ameliorate DKD through the activation of receptors and downstream signaling pathways in the glomerular cells. The farnesoid X Receptor (FXR) pathway and takeda G protein-coupled receptor 5 (TGR5), which are directly activated by BAs, are highly expressed in the kidney after activation and can play a role in slowing down renal injury (90–94). However, the association between BAs and DKD remains unclear. (**Figure 1**)

## 3 Mechanisms of lipid metabolic changes in DKD

### 3.1 Metabolic reprogramming (MR)

MR refers to the ability of cells to adapt their metabolic processes in response to changing environmental conditions (95) and MR in DKD mainly manifests as renal lipid accumulation (96). Among these, abnormal metabolic pathways of TG, CHOL, sphingolipids, LDs, and BAs are key aspects of MR in DKD.

Renal TG accumulation in patients with DKD is associated with the dysregulated expression of genes involved in lipid metabolism (97). Renal biopsies from patients with DKD showed decreased expression of genes encoding PPAR-α and PPARδ and their downstream acyl-coenzyme A oxidase and carnitine palmitoyl transferase (CPT1) involved in the fatty acid β-oxidation pathway, and SREBP, a transcription factor regulating FA synthesis, induced fluorescent antibody serum neutralization (FASN) and acetyl Coenzyme A(CoA) carboxylation. The expression of genes involved in the fatty acid β-oxidation pathway, such as (22),and SREBP, a transcription factor regulating the synthesis of FAs, induced FASN and acetyl CoA carboxylase to increase the cytosolic TG content (98, 99),and it was found that Streptozotocin(STZ)-induced diabetic rat renal cortex and DKD patients’ renal tubules increased their TG content and increased sterol regulatory element-binding proteins 1(SREBP-1) expression in the renal tubular epithelium of STZ-induced diabetic rats (100–103). In addition, elevated renal TC was also associated with decreased PPAR-α and PPAR-δ expression, which also led directly to decreased FAO (44), showing a direct pathway between decreased FAO and net accumulation of lipids in the renal cortex of patients with DKD.

In CHOL metabolism, low-density lipoprotein receptor (LDLr) and 3-hydroxy-3-methylglutaryl-CoA (HMG-CoA) were involved in CHOL uptake and synthesis, respectively. The expression of LDLr and HMG-CoA reductase was significantly elevated in DKD, whereas the expression of genes involved in CHOL efflux, including ABCA1, ATP-binding cassette transporter G1(ABCG1), and apoipoprotein E (apoE), was significantly reduced (38, 104, 105). Moreover, sterol regulatory element-binding proteins 2(SREBP-2) activates LDLr and HMG-CoA reductase, enhancing CHOL uptake and synthesis (46, 106). ABCA1 mediates CHOL transport to apolipoprotein A-I (Apo A-I) for further efflux, and strong downregulation of ABCA1 mRNA was observed in DKD, leading to the inhibition of CHOL efflux in pedicle cells (52, 107).

In terms of sphingolipids metabolism, the rs267734 gene variant of Ceramide synthases 2 (CerS2) in patients with DKD resulted in increased proteinuria (108), and polymorphisms in the Sphingosine-1-phosphate lyase 1(SGPL1) gene encoding S1P lyase 1 were associated with reduced enzymatic activity of S1P lyase 1 and the development of nephropathy. In mice, knockdown of the Sgpl1 gene encoding S1P lyase 1 resulted in loss of peduncles and severe proteinuria (109, 110). Studies on C1P have shown that increased SMPDL3b expression in DKD mice is associated with podocyte C1P deficiency (62). SMPDL3b expression is elevated in the glomeruli of patients with DKD (63), whereas SMPDL3b overexpression in podocytes leads to S1P accumulation (64).

Accumulation of LDs in DKD may be related to abnormal protein expression in the coating of LDs. It was found that variations in Perilipin 1(PLIN1) can lead to DKD-like renal injury (111). Clinical studies have shown that the polymorphism rs4578621 in the Perilipin(PLIN) gene is associated with type 2 diabetes mellitus, and the expression of Perilipin 2(PLIN2) is upregulated in the kidneys of diabetic db/db mice (103, 112),which may be the reason.

**Figure 2:**
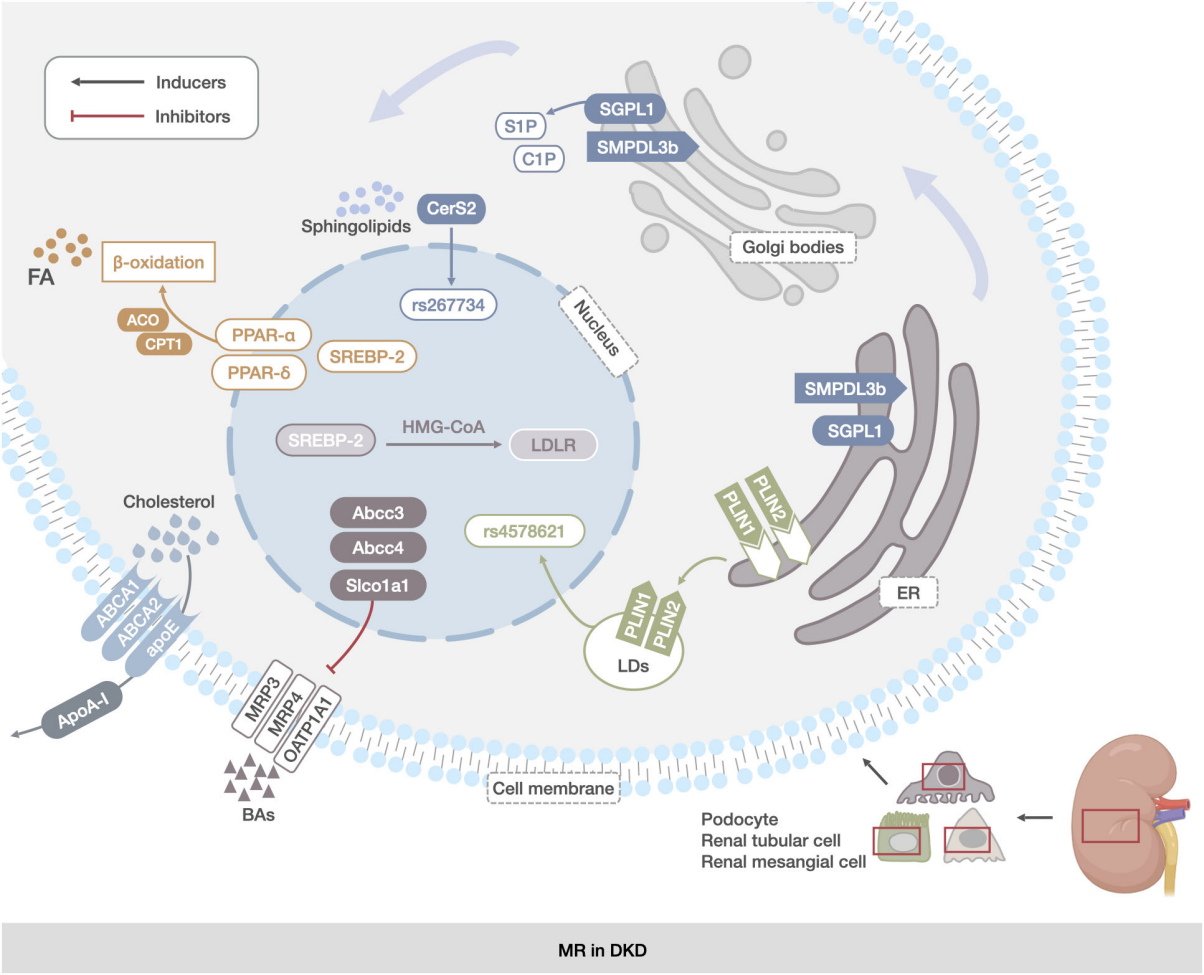
MR in DKD. The main manifestations were abnormal reprogramming of TG, CHOL, sphingolipids, LDs, and BAs metabolic pathways in renal mesangial cells, renal tubular cells, and podocytes. TG abnormalities were associated with increased expression of FA synthesis transcription factors and decreased expression of proteins of the fatty acid β-oxidation pathway (PPAR-α, PPAR-δ, and SREBP). The abnormalities in CHOL metabolism were related to the abnormal expression of genes encoding CHOL uptake and synthesis proteins (ABCA1, ABCG1, and apoE).Abnormalities in sphingolipids metabolism were associated with deletion of the SGPL1 gene and increased expression of the SMPDL3b protein. Changes in LDs were associated with increased expression of PLIN2. Changes in BAs metabolism were associated with increased expression of the genes encoding BAs transporter proteins (Abcc3, Abcc4, and Slco1a1).

In BA metabolism, the ATP-binding cassette transporter C 3 (Abcc3) encodes multidrug resistance-associated protein 3 (MRP3), and Abcc4 encodes MRP4. Both of transport taurine and glycine conjugates of bile acids and unconjugated bile acid cholate into the bloodstream. Solute carrier organic anion transporter family member 1A1 (Slco1a1) encodes organic anion transport peptide 1A1 (OATP1A1), which transports unconjugated and conjugated bile acids into the cell (113),and type 2 diabetic db/db mice exhibit decreased Slco1a1 and increased Abcc3 and Abcc4 expression in the kidney, resulting in the loss of bound and unconjugated bile acids and bile salts from the cells (114, 115). (**Figure 2**)

### 3.2 Ferroptosis

Lipid metabolism disorders in DKD are primarily characterized by disturbances in FA, BA, and CHOL metabolism (116, 117). DKD serum is markedly decreased in l-methionine, which can be methylated *in vivo* to produce L-(+)-cysteine. The latter is one of the three amino acids used for the synthesis of glutathione (GSH) (118). Elevated Fatty Acid Binding Protein 4(FABP4) expression may lead to altered lipid deposition in DKD and is associated with ferroptosis (119). Elevated expression of FABP4 was found in HG-HK2 cells from patients with DKD who showed iron deposition in renal tubules and loss of mitochondrial cristae, whereas Carnitine Palmitoyl transferase 1A(CPT1A), glutathione peroxidase 4, ferritin heavy chain (FTH), and ferritin light chain (FTL) were found to be elevated in HG-HK2 cells from patients with DKD who showed iron deposition in renal tubules and loss of mitochondrial cristae. FTH and FTL decrease and promotes ferroptosis, leading to renal tubular injury. Simultaneous inhibition of FABP4 restores FAO, thereby reducing lipid accumulation and peroxidation while increasing CPT1A expression, which, in turn, inhibits ferroptosis and reduces renal injury and fibrosis (120). Acyl-CoA synthetase long-chain family4 (ACSL4) is overexpressed in DKD (121), and FA regulation modulates lipid metabolism to affect ferroptosis. The upregulation of ACSL4 increased Arachidonoyl-Phosphatidylethanolamine (AA-PE) and Adrenoyl-Phosphatidylethanolamine (AdA-PE) levels, promoted ferroptosis, and exacerbated tubular fibrosis in DKD (122). In addition, ACSL4 inhibition may ameliorate renal injury by decreasing the levels of lipid peroxidation products and inhibiting ferroptosis (123). CD36 expression is increased in patients with DKD (124). CD36 transports polyunsaturated fatty acids (PUFAs), which are essential for lipid peroxidation, intracellularly, and its increased expression has been shown to correlate with ROS production (1). CD36 has been shown to promote proximal tubular fibrosis under hyperglycemic conditions, which may be mediated by its regulation of ferroptosis suppressor function in the proximal tubular cells. CD36 has been shown to promote proximal tubular fibrosis under hyperglycemic conditions. This may promote ferroptosis by regulating the ubiquitination of ferroptosis suppressor protein 1 in proximal tubular cells (125, 126). A correlation has been found between BA metabolism and glomerulosclerosis and tubulointerstitial fibrosis in DKD (127, 128), possibly through the inhibition of ligand-activated nuclear receptor Farnesoid X Receptor(FXR)/retinoid X receptor activation, which is strongly associated with ferroptosis (129). In addition, compared to soybean oil (SO) and linoleic acid (LN), which are rich in PUFAs, peanut oil (PO), lined oil (LO), and rapeseed oil (RO), which are rich in saturated fatty acids and monosaturated fatty acids, are highly likely to reduce the reabsorption of BAs in the colon, which has a different impact on BA metabolism. This may be related to changes in the gut microbiota structure of DKD (130). Impairment of CHOL efflux and the accumulation of CHOL lead to glomerulosclerosis and podocyte ferroptosis in early DKD, and is related to the reduction of ABCA1, the main protein of CHOL efflux (131, 132). The interaction between CHOL metabolism and ferroptosis (133, 134) may be a potential cause of DKD progression, suggesting that ferroptosis is correlated with FAs, BAs, CHOL metabolism disorders, and DKD development.

### 3.3 Lipophagy

Autophagy is an intracellular pathway that maintains cellular homeostasis by degrading cytoplasmic components via autolysosome formation of autolysosomes (135). Studies have shown that the development of DKD is associated with defective renal autophagy (119, 136–140). In DKD, the microtubule-associated protein 1A/1b-light chain 3 (LC3-II) is dependent on phagocytosis of endoplasmic reticulum membranes to form lipid autophagosomes, which with their cargoes, mainly composed of ChE and TG, fuse with lysosomes to form autophagic lysosomes, in which the cargo is degraded to produce FAs, a process known as lipophagy (141–147). The accumulation of ChE and FAs metabolites in DKD podocytes has been implicated in the pathogenesis of glomerular dysfunction and lipotoxicity in DKD (148). LDs, as reservoirs of excess lipids, inhibit lipotoxicity, and over-activation of lipophagy can promote renal fibrosis (81, 149). Adipose triglyceride lipase (ATGL) is a critically important signaling node for lipophagy, and sirtuin 1 (SIRT1) acts as a key mediator downstream of ATGL whose role is to promote lipophagy (150) and decreased expression of SIRT1 in the kidney promotes DKD (151–153). In addition, LDs are subject to a variety of cellular factors that can influence the development of lipophagy. Lipophagy is regulated by the nutritional state of the cell and proteins that detect changes in the nutritional stores (154). Mechanistic target of rapamycin complex 1(mTORC1) inhibits lipophagy, and activation of AMP-activated protein kinase (AMPK) promotes lipophagy (155, 156). Specific activation of mTORC1 in the podocytes in DKD leads to many changes in DKD, including increased albuminuria, podocyte loss, and thylakoid membrane expansion, while AMPK in DKD decreased autophagic activity in podocytes and increased cytotoxicity and apoptosis (157, 158).

Sphingolipids may be a regulator of lipophagy (159). The sphingolipids metabolite C1P also regulates renal autophagy (160, 161). Cer itself induces autophagy, and treatment with exogenous C1P can upregulate the expression of beclin1, leading to autophagy through c-Jun N-terminal kinase (JNK) activation (162), whereas AMPK can initiate autophagy either by phosphorylating beclin1 or by blocking mTORC1 (163, 164). This indicates an important role for the AMPK/mTOR pathway in autophagy. CHOL removal also plays a crucial role in autophagosome initiation (165, 166). It was found that STZ induced a decrease in autophagic activity in podocyte cells after diabetes, which ultimately led to the development of DKD (167), and the abnormalities of sphingolipids and CHOL exhibited by lipid metabolism disorder in patients with DKD may be responsible for the inhibition of autophagy in DKD podocyte cells. The above suggests that in DKD, lipophagy abnormalities and autophagy inhibition caused by sphingolipids and CHOL metabolism disorders are closely related to the development of DKD.

### 3.4 Immunomodulation of gut microbiota

Gut microorganisms produce metabolites, such as short-chain FAs (SCFAs), which are involved in the synthesis and metabolism of the human body and in the immunomodulatory processes of the body (168, 169). The gut microbiota influences both innate and adaptive immune systems. During innate immunity, the gut microbiota of *Bacteroides, Bifidobacterium, Lactobacillus*, and *Aspergillus* are involved in the maturation of the immune system (170). SCFAs, metabolites of gut microbiota, are involved in immunomodulation by regulating nuclear factor kappa-B (NF-кB) signaling in neutrophils, eosinophils, and macrophages in the gut and by strengthening the physical barrier of the gut (171–174). During adaptive immunity, *Lactobacillus, Clostridium, Bifidobacterium*, and *Enterococcus* in the gut can reduce inflammatory responses by producing lipid metabolites and reducing tumor necrosis factor α (TNF-α), and inflammatory mediators interleukin-1(IL-1), interleukin-6 (IL-6), and interleukin-18(IL-18) (175–178).

**Figure 3:**
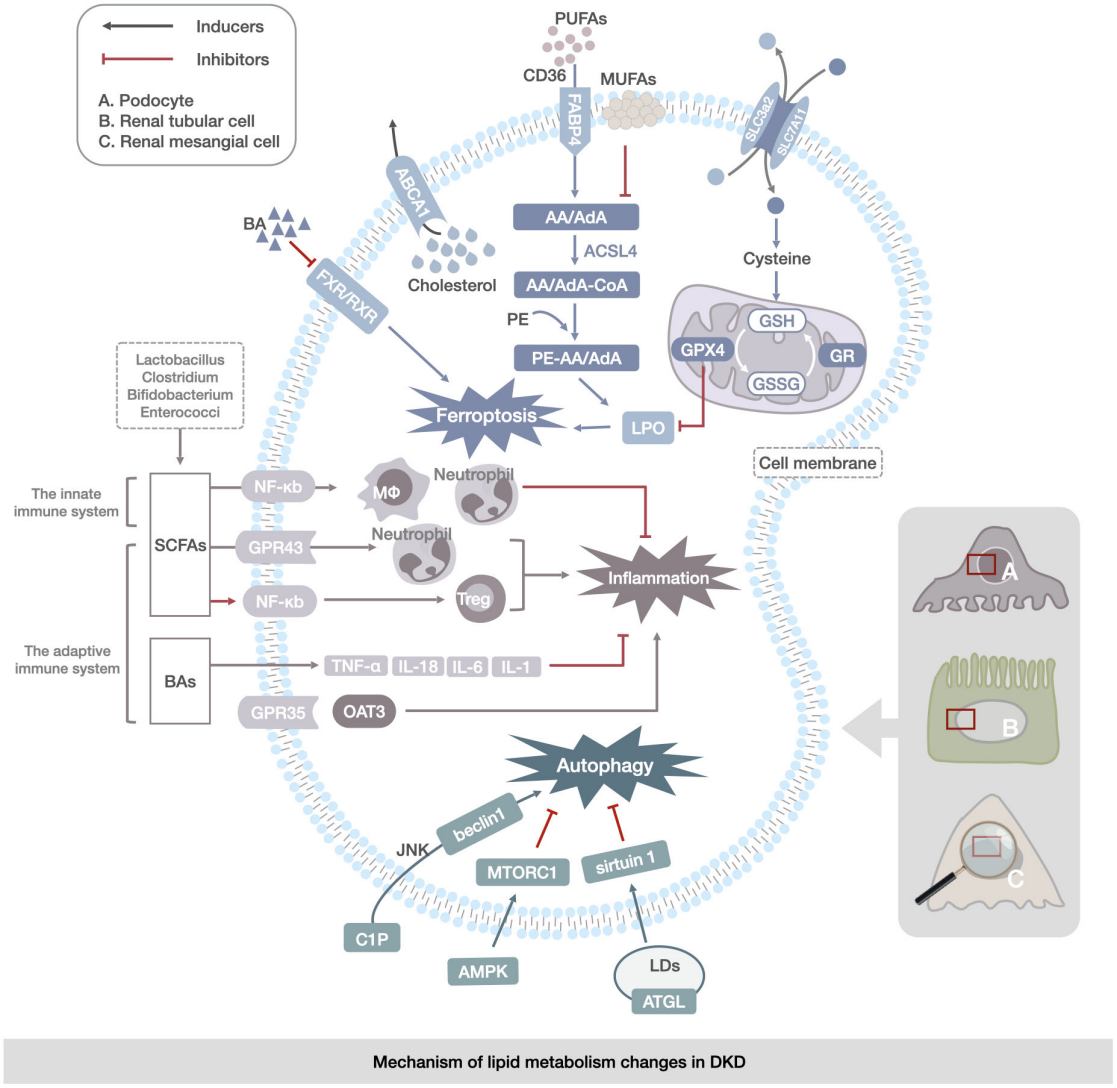
Mechanism of lipid metabolism changes in DKD. The main manifestations are abnormalities of ferroptosis, lipophagy, and immunoregulation of gut microbiota in renal mesangial cells, renal tubular cells, and podocytes. Ferroptosis abnormalities are reflected in the transport of PUFAs and changes in the enzymatic response to the LPO process. Abnormalities in lipophagy are associated with abnormalities in cytosolic C1P and CHOL metabolism, leading to the regulation of autophagy by the JNK/AMPK/mTOR channel activation to regulate autophagy in LDs. Abnormalities in gut microbiota immunoregulation are reflected in disorders of gut-derived SCFAs, BAs, and immune factors (TNF-α,IL-1, IL-6, and IL-18) via the NF-кB, OAT3 pathway directly contributing to renal inflammation and lipid accumulation in DKD.

In DKD, the gut microbiota are associated with immune dysregulation, lipid metabolism disorders, and DKD development (172–174, 179, 180). The gut microbiota of *Lactobacillus, Clostridium, Bifidobacterium*, and *Enterococcus* (175) can control BAs as lipid metabolism modulators to modulate the adaptive immunosuppression of inflammatory responses by altering CHOL secretion (177, 178). The gut-liver-kidney axis is the pathway by which lipid metabolites are metabolized in the liver through intestinal absorption and excreted from the kidney (181). Organic anion transporter 3(OAT3) is mainly expressed in the kidney, and the absence of OAT3 alters the normal metabolite transport function in the gut-liver-kidney axis, resulting in the accumulation of endogenous lipid metabolites, such as bile acids and lipids, and G-protein-coupled receptor 35(GPR35), which is a key receptor for lipid metabolism. The coupled GPR35 is associated with inflammation (182–184). This suggests that the disturbance of the gut microbiota and imbalance of immune homeostasis in patients with DKD may affect lipid metabolism through the gut-liver-kidney axis and ultimately contribute to the development of DKD (**Figure 3**).

## 4 Targeting lipid metabolism for DKD treatment

### 4.1 Conventional drugs

#### 4.1.1 Atorvastatin

Atorvastatin effectively reduced the levels of low-density lipoprotein CHOL (LDL-C), creatinine (CREA), and urinary albumin and creatinine (UACR) and downregulated the expression of the inflammatory factors TNF-α, monocyte chemoattractant protein-1 (MCP-1), and IL-6 expression in renal tissues, which ameliorates renal injury and delays the progression of DKD by reducing morphological lesions and renal fibrosis and increases transforming growth factor beta (TGF-β) and collagen I staining (185).

#### 4.1.2 Fenofibrate

Fenofibrate decreased TG content and lipid accumulation in DKD and increased activation of the AMPK/FOXA2/medium-chain acyl-CoA dehydrogenase pathway, significantly reducing renal function and tubular cell apoptosis and slowing DKD progression (186).

#### 4.1.3 Betulinic acid (BA)

BA inhibits phospho-inhibitor of kappa Balpha (IκBα) degradation and NF-κB activity and reduces Fibronectin (FN) expression. It inhibited the DNA-binding activity and transcriptional activity of NF-κB in high glucose-induced glomerular mesangial cells, enhanced the interaction between IκBα and β-arrestin 2 in mesangial cells, and prevented diabetic renal fibrosis by stabilizing the NF-κB inhibitory protein, IκBα, to inhibit NF-κB activation (187).

#### 4.1.4 Liraglutide

Liraglutide is a novel hypoglycemic drug. Inhibition of SREBP-1 and Fatty Acid Synthase (FAS) increases ATGL and hormone-sensitive lipase protein expression levels, promoting AMPK phosphorylation to attenuate ectopic lipid deposition in renal tubules, improving PA-induced lipid accumulation in renal tubular epithelial cells, inhibiting lipid synthesis, and promoting lipolysis (188). Liraglutide increased the expression of phosphorylated (p)-eNOS and p-AMPK in the glomeruli, downregulated the expression of p-mTOR, increased the renal expression of LC3B-II, activated autophagy, ameliorated DKD kidney injury, and decreased urinary albumin and Liver-type Fatty Acid Binding Protein (L-FABP) levels (21).

#### 4.1.5 α-lipoic acid (ALA)

ALA plays a role as an antioxidant in the mitochondrial dehydrogenase reaction, which improves the antioxidant status and lipid distribution, and reduces inflammation by regulating lipid levels, enhancing the body’s antioxidant capacity, protecting vascular endothelial function, and activating the renal cystathionine gamma-lyase/hydrogen sulfide pathway to delay DKD (189).

#### 4.1.6 Adiponectin Receptor Agonist AdipoRon

AdipoRon is an active synthetic lipocalin receptor agonist. AdipoRon ameliorates DKD by activating the intracellular Ca2+/Liver Kinase B1(LKB1)-AMPK/PPARα pathway to ameliorate glomerular endothelial cells(GECs) and podocyte injury (190). AdipoRon reduces palmitate-induced lipotoxicity in the kidney by improving lipid metabolism, especially in GECs and podocytes, and reduces oxidative stress and apoptosis, and preventing renal injury, thereby improving endothelial dysfunction and delaying DKD progression in type 2 diabetic nephropathy (191).

#### 4.1.7 Apolipoprotein A-IV (apoD) and apolipoprotein D (apoA-IV)

APOD is an essential component of plasma lipoproteins and plays an important role in plasma lipoprotein metabolism. Increased apoD and apoA-IV help counteract the chemical modification of high-density lipoprotein (HDL) by advanced glycation end products (AGEs) and carbamylation, which contributes to the loss of function of HDL in maturing DKD, thereby delaying DKD (192).

#### 4.1.8 Metrnl

Metrnl is a recently discovered hormone produced by skeletal muscles and adipose tissue in response to exercise and cold exposure. Metrnl-specific overexpression or recombinant Metrnl administration in the kidney regulates renal tubular lipid metabolism through mitochondrial homeostasis mediated by the Sirtuin 3(Sirt3)-AMPK/uncoupling protein 1(UCP1) signaling axis, alleviates renal injury, and delays DKD in diabetic mice (193).

#### 4.1.9 Lipin-1

Lipin-1 inhibits adipose synthesis, upregulates FAO, attenuates proximal tubular epithelial cell injury in tubulointerstitial fibrosis, and delays DKD by promoting proliferator-activated receptor-gamma co-activator-1alpha(PGC-1α)/PPARα-mediated Carnitine Palmitoyltransferase 1 Alpha(Cpt1α)/hepatocyte nuclear factor 4alpha signaling and upregulating SREBPs (194).

#### 4.1.10 Leptin

Leptin is a 167-amino acid lipoprotein that plays a role in the regulation of energy metabolism. It attenuates lipid deposition present in the kidney by activating AMPK phosphorylation, which upregulates insulin-induced gene 1 (Insig-1) expression in PA-induced renal tubular epithelial cell lines(NRK-52E) and delays DKD (195).

#### 4.1.11 ABCA1

ABCA1 is one of the most important proteins involved in the maintenance of CHOL homeostasis. In the human renal glomerular endothelial cell line cultured under high-glucose and high-CHOL conditions, ABCA1 deficiency increases cellular CHOL deposition, leads to inflammation and apoptosis, disrupts the endothelial glycoconjugate barrier, and induces endoplasmic reticulum stress (ERS). In contrast, ABCA1 overexpression enhances CHOL efflux or inhibits ERS *in vitro*, significantly prevents high glucose- and high-CHOL-stimulated glomerular endothelial injury, and delays DKD (196).

#### 4.1.12 Maresin 1 (MaR1)

MaR1 is a widespread anti-inflammatory lipid mediator, and serum MaR1 concentrations are negatively correlated with hemoglobin A1c, diabetes duration, UACR, neutrophils, and the neutrophil-lymphocyte ratio and positively correlated with HDL CHOL (HDL-C) and estimated glomerular filtration rate. MaR1 alleviated the pathological progression of hyperglycemia, UACR, and DKD through the leucine-rich repeat domain-containing G protein-coupled receptor 6(LGR6)-mediated cyclic adenosine monophosphate-superoxide dismutase-2 antioxidant pathway (197).

#### 4.1.13 Exogenous Adropin (Ad) in nanocapsules

Ad reverses the effects of nanocapsules on ameliorating mitochondrial damage by knocking down the overexpression of Neuronatin (Nnat) or translocator protein(TSPO) to improve lipid metabolism and inhibit TSPO activity, thereby enhancing mitochondrial function. It protects hexokinase 2(HK2) from high glucose (HG) stimulation. It also effectively controls blood glucose and lipid levels, improves renal function, inhibits ROS overproduction, protects mitochondria from damage, improves lipid deposition in renal tissues, and downregulates the expression of lipogenic proteins SEBP-1 and Adipose Differentiation-Related Protein (ADRP) in DKD mice (198).

#### 4.1.14 Novel phosphate and bile acid storage agent polymer SAR442357

SAR442357 is a newly developed non-absorbable polymeric sequestering agent with optimal phosphate and bile salt sequestration properties. Long-term treatment of diabetes mellitus type 2(T2DM) obese Zucker fatty/spontaneously hypertensive heart failure F1 hybrid (ZSF1) leads to enhanced segregation of BAs and phosphates in the gut, improved glycemic control, reduced serum CHOL, and delayed DKD progression (199).

#### 4.1.15 Complement factor B knockout (Cfb-knockout)

Effects of Complement Factor B(CFB) on lipid metabolism in developing DKD, Cfb-knockout diabetic mice had significantly less vas deferens interstitial injury and less Cer biosynthesis. Cfb knockout further blocked the transcription of Ceramides (CERs) by inhibiting the NF-κB signaling pathway, which inhibited the activation of the complement alternative pathway and attenuated renal injury in DKD, especially vas deferens mesenchymal injury. CERs regulated the biosynthesis of Cer (200). (**Table 1**)

**Table 1:** Therapeutic advances related to conventional drug-targeted lipid metabolism in diabetic kidney disease.

Categorization	Veterinary drug	Experimental model	Signaling pathway	Mechanism of action	Related Literature
Antihyperlipidemic drug	Atorvastatin	STZ rats		TNF-α, MCP-1 and IL-6 downregulated	(185)
	Fenoprofibrate	db/db mice; PA/HG-induced HK2 cells	AMPK/FOXA2/MCAD pathway		(186)
	BA	STZ-induced diabetic rats; HG-induced GMCs	Inhibition of NF-κB activation	Stabilization of the NF-κB inhibitory protein IκBα	(187)
Antihyperglycemic drug	Liraglutide	SDT rats		(p)-eNOS and p-AMPK upregulated; p-mTOR downregulated; LC3B-II expression increased	(188)
		Male SD rats were treated with a combination of high-fat diet + unilateral nephrectomy + low-dose STZ in order to establish a rat model of DN	AMPK phosphorylation	SREBP-1 and FAS inhibition; ATGL and HSL upregulation	(21)
Improve blood vessels	ALA	Nicotinamide/STZ SD rats	Renal CSE/H2S pathway		(189)
New drug	The Adiponectin Receptor Agonist AdipoRon	db/db mice	Ca2+/LKB1-AMPK/PPARα pathway	Upregulation of CaMKKβ, phosphorylated Ser431LKB1, phosphorylated Thr172AMPK and PPARα expression	(191)
	apoD and apoA-IV	DKD patients		HDLand AGEs downregulated	(192)
	Metrnl	HFDs/STZ-induced mice; db/db mice	Sirt3-AMPK/UCP1 signaling axis		(193)
	Lipin-1	Lipin-1-deficient db/db mouse model and STZ/HFD-induced T2DM mice	PGC-1α/PPARα-mediated Cpt1α/HNF4α signaling	SREBPs upregulated	(194)
	Leptin	STZ rats	AMPK phosphorylation	Insig-1 expression is upregulated in NRK-52E cells	(195)
	ABCA1	T2DM mice		Enhanced CHOL efflux; ERS inhibition	(196)
	MaR1	Patients with glucose tolerance/T2DM/DKD; STZ vs. HFD mice	LGR6-mediated cAMP-SOD2 antioxidant pathway		(197)
	Ad		knocking down the overexpression of Nnat or TSPO	inhibits ROS overproduction;, SEBP-1 and ADRP downregulated	(198)
	SAR442357	ZSF1 rats		enhanced segregation of BAs and phosphates,;improved glycemic control, reduced serum CHOL	(199)
Gene therapy	Cfb-knockout	DN patients	NF-κB signaling pathway	CERS transcriptional down-regulation	(200)

### 4.2 Traditional Chinese medicine (TCM) monomers and compound formulas

#### 4.2.1 Lipid metabolism reprogramming

##### 4.2.1.1 Berberine (BBR)

BBR is a potent compound from TCM (201) that can reverse lipid metabolism disorders and ameliorate kidney injury in patients with DKD. BBR stabilizes mitochondrial morphology in podocytes by eliminating PA-induced activation with dynamin related protein 1 (202).BBR peroxisome Peroxisome Proliferator-Activated Receptor Gamma Coactivator 1-Alpha(PGC-1α) signaling pathway activation promotes mitochondrial energetic homeostasis and FAO in podocytes, and PGC-1α-mediated mitochondrial bioenergetics can play a key role in lipid disorder-induced podocyte injury and development of DKD in mice (203).

##### 4.2.1.2 Breviscapine

Breviscapine is a purified flavonoid extract of Erigeron breviscapus (204), which attenuates dyslipidemia by decreasing 24-h urine protein, serum creatinine (Scr),and blood urea nitrogen levels; modulates lipid profiles by increasing levels of TC, TG, and HDL; and protects against kidney injury (205).

##### 4.2.1.3 Microvascular endothelial differentiation gene-1 (MDG-1)

MDG-1 is a polysaccharide derived from TCM japonicas (206). MDG-1 reduced blood glucose, TG, Blood Urea Nitrogen (BUN), and albumin levels by activating the phosphatidylinositol-3 kinase/Akt signaling pathway and significantly suppressed the expression of TGF-β1 and connective tissue growth factor. MDG-1 attenuated glomerular mesangial dilatation and tubulointerstitial fibrosis in diabetic mice. MDG-1 ameliorated DKD by reducing hyperglycemia, hyperinsulinemia, and hyperlipidemia and by inhibiting intracellular signaling pathways (207).

#### 4.2.2 Ferroptosis

##### 4.2.2.1 Proteoglycan FYGL

FYGL is a water-soluble substance extracted from *Ganoderma lucidum*, highly branched proteoglycan that protects tissues from oxidative stress damage (208). FYGL significantly inhibited HG/PA-induced proliferation of HBZY-1 cells, ROS generation, and malondialdehyde (MDA) production; promoted Superoxide Dismutase(SOD) activity; and suppressed the expression of NADPH oxidase 1(NOX1), NADPH oxidase 4(NOX4), mitogen-activated protein kinase, NF-κB, and pro-fibronectin expression. It significantly alleviates lipid metabolism disorders and protects the kidneys from oxidative stress-induced dysfunction, delaying DKD (209).

##### 4.2.2.2 Notoginsenoside R1 (NGR1)

NGR1 is a novel saponin from Panax notoginseng, a TCM for the adjuvant treatment of DKD (210),which demonstrated that NGR1 treatment increased serum lipids in db/db mice, reduced AGE-induced mitochondrial damage, limited the increase in ROS, reduced apoptosis in HK-2 cells, promoted the expression of nuclear factor erythroid 2-related factor 2(Nrf2) and heme oxygenase-1(HO-1) to abrogate apoptosis-inducing and TGF-β signaling by ROS, attenuated histological abnormalities in the kidney, reduced glomerular volume in DKD, inhibited oxidative stress-induced apoptosis and renal fibrosis, and delayed DKD (211).

##### 4.2.2.3 Triptolide

The TCM *Tripterygium wilfordii* (TWH) has been used clinically to treat renal diseases (212). Triptolide, the main active ingredient of TWH, can reduce the 24-h urine total protein quantity (24-h UTP), resulting in decreased renal MDA and nitrotyrosine expression, downregulation of renal oxidative carbonyl protein (OCP) expression, and elevated renal SOD to delay DKD (213). In addition, triptolide is an active diterpene purified from the TCM TWH, which can ameliorate hyperlipidemia and albuminuria in db/db diabetic mice, alleviate glomerular hypertrophy and pedunculated cell injury, and attenuate inflammation and oxidative stress in the kidneys (214).

##### 4.2.2.4 Mulberry extract

Mulberry extract is considered a potential therapeutic drug for diabetes (215). Mulberry extract can lead to a significant reduction in serum TG and very low-density lipoprotein CHOL and HDL-C concentrations, improve plasma GSH and Malondialdehyde (MDA), and delay the development of DKD (216).

#### 4.2.3 Lipophagy

##### 4.2.3.1 Panax japonicus C.A. Meyer (PJ)

PJ has been shown to exert a therapeutic effect on DKD (217). PJ can reduce hyperlipidemia, serum BUN, and 24-h UTP in diabetic mice by modulating unsaturated FAs, glycerophospholipid metabolism, and purine metabolism; protect against pathological changes in renal tissues; and prevent apoptosis of renal cells by modulating the beclin-2/caspase 3 apoptosis signaling pathway to delay DKD (218).

##### 4.2.3.2 Resveratrol (RSV)

RSV, an extract of the Chinese herb Tiger Balm, is a naturally occurring polyphenolic compound that reduces blood glucose and lipid concentrations and is a Sirt1 agonist (219). Resveratrol improves circulating lipids and renal dysfunction, reduces lipid deposition in the kidney by modulating the junctional adhesion molecule-like protein/Sirt1 lipid synthesis pathway, and ameliorates DKD (220).

#### 4.2.4 Immunomodulation of gut microbiota

##### 4.2.4.1 Cordyceps cicadae polysaccharides (CCP)

CCP is a fungus that parasitizes ghost moth larvae, modulates lipids, and improves DKD (221). CCP increases *Lactobacillus* and *Anaplasma* community abundance while decreasing the abundance of LPS-producing bacteria and reducing the levels of serum TNF-α, IL-1β, and IL-6 in mice. Significant improvements in 24-h urine output levels and urinary protein, albumin to creatinine ratio, and Scr levels and a decrease in glomerular mesangial zone collagen fibers and lipid accumulation were observed in renal tissue samples (222).

##### 4.2.4.2 Magnesium lithospermate B (MLB)

MLB, an aqueous extract of *Salvia miltiorrhiza*, an erect perennial herb of the genus Salvia and family Labiatae, is a potential therapeutic agent for kidney disease (223). The abundance of *Shigella* and *Aspergillus* species and fecal BAs levels in the rat intestine were significantly reduced by MLB intervention, suggesting that MLB may restore the integrity of the intestinal barrier and inhibit the release of BA-induced inflammatory cells through localized modulation of the gut microbiota and BA metabolism, slowing renal injury (224). (**Table 2**)

**Table 2:** Therapeutic advances related to herbal targeting of lipid metabolism in diabetic kidney disease.

Categorization	Veterinary drug	Base	Experimental model	Signaling pathway	Mechanism of action	Related Literature
Lipid metabolism reprogramming	BBR	Coptis chinensis	db/db mice	PGC-1α signaling pathway		(202)
			HFD/STZ-induced DKD rats	Protein expression of GRKs in the G protein-AC-cAMP signaling pathway	GRK2, GRK3 downward; GRK6 upward	(203)
	Breviscapine.	Erigeron breviscapus (Vant.) Hand.-Mazz	A meta-analysis of patients with RCT and DN is shown		decreasing 24-h urine protein, Scr and blood urea nitrogen levels; increasing levels of TC, TG, and HDL	(205)
	MDG-1	Ophiopogon japonicus	KKAy mice	PI3K/Akt signaling pathway	Decreased levels of blood glucose, TG, BUN, and albumin;suppressed expression of TGF-β1 and connective tissue growth factor	(207)
Ferroptosis	FYGL	G. lucidum	db/db mice; HG/PA-induced rat glomerular body mesangial cells (HBZY-1)		HBZY-1 cell proliferation,;ROS, MDA inhibition; SOD upregulation;Inhibition of NOX1, NOX4, MAPK, NF-κB, and Fibronectin Expression	(209)
	NGR1	Panax notoginseng	AGEs-induced db/db mice; HK-2 cells		Nrf2 and HO-1 expression promotes	(211)
	Triptolide	Tripterygium wilfordiiHook.f., TWH	STZ rats		24-h UTP,MDA, nitrotyrosine, OCP downregulated; SOD upregulated	(213, 214)
	Mulberry extract	Mulberry	DKD patients		GSH and MDA downregulated	(216)
Lipid autophagy	PJ	Panax japonicus C.A. Meyer	HFD/STZ-induced diabetic mice	Bcl-2/caspase 3 apoptosis signaling pathway		(218)
	RSV	Tiger Balm	Patients with type 2 diabetes and albuminuria	JAML/Sirt1 lipid synthesis pathway	reduces lipid deposition	(220)
Immunomodulation of intestinal flora	CCP	ghost moth larvae	mice		TNF-α, IL-1β, IL-6 downregulated; ACR and Scr upregulated	(222)
	MLB	Salvia miltiorrhiza	rats		BAs downregulated	(224)

## 5 Summary

The altered manifestations of lipid metabolism disorder present in DKD have been gradually clarified, and the mechanisms affecting this process mainly include cellular ferroptosis, lipophagy, reprogramming of lipid metabolism, and immune modulation of the gut microbiota (involving the liver-kidney axis) in the body, which, in turn, accelerates the progression of DKD, a process that suggests one of the relationships between lipid metabolism disorder and DKD. Recent research has suggested that interventions targeting altered lipid metabolism disorder may help improve DKD prognosis. However, this requires further clarification of the specific targets of the interventions; otherwise, the interventions may not be effective. In addition, compared with known lipid-lowering drugs, natural drugs have the advantage of multiple targets and multiple pathways in the treatment of DKD, but their mechanism of action and scope of application have not yet been clarified. Most of the existing studies have focused on the monomers of TCM, whereas the complexity of the components of TCM prescriptions commonly used in the clinic makes it difficult to elucidate the mechanism of action. The role of lipid-lowering as a target of natural drug action warrants further study of the relevant mechanisms between DKD and these two diseases and provides a potential research direction for the effective treatment of DKD.

Computer modeling and simulation technologies are now pivotal in identifying therapeutic targets for diseases like cancer, liver fibrosis, and Takotsubo syndrome (TTS) (225–230). Future studies can leverage cost-effective methods like quantitative lipid analysis with ES-MSI and incorporate molecular dynamics (MD) simulations alongside computational bioinformatics to investigate lipid metabolism’s role in diabetic kidney disease (DKD) and natural drug effects on lipid disorders. Starting with MD simulations for atomic-level molecular interaction insights, crucial for DKD’s molecular understanding, followed by computational tools to analyze complex data for genetic and protein stability patterns, this approach aims to elucidate lipid metabolism’s link to DKD and refine therapeutic targets by analyzing lipid profile shifts and SNPs.

This streamlined, multidisciplinary approach promises a deeper understanding of DKD’s pathophysiology and treatment, underscoring the importance of merging computational and experimental methods in biomedical research to enhance knowledge and therapeutic developments.

## Author contributions

Y-ZH: Writing – review & editing, Writing – original draft, Validation, Supervision, Project administration, Methodology, Investigation, Formal analysis, Data curation, Conceptualization. B-XD: Writing – original draft, Methodology, Data curation. X-YZ: Writing – original draft, Methodology, Data curation. Y-Z-YW: Writing – original draft, Methodology. H-JZ: Writing – review & editing, Validation, Supervision, Resources, Project administration, Methodology, Funding acquisition, Conceptualization. W-JL: Writing – review & editing, Validation, Supervision, Resources, Methodology, Funding acquisition, Conceptualization.

## References

[1] Alicic RZ Rooney MT Tuttle KR. Diabetic kidney disease: challenges, progress, and possibilities. Clin J Am Soc Nephrol. (2017) 12:2032–45. doi: 10.2215/CJN.11491116 PMC5718284 28522654

[2] Thomas MC Brownlee M Susztak K Sharma K Jandeleit-Dahm KA Zoungas S. Diabetic kidney disease. Nat Rev Dis Primers. (2015) 1:15018. doi: 10.1038/nrdp.2015.18 27188921 PMC7724636

[3] Tuttle KR Agarwal R Alpers CE Bakris GL Brosius FC Kolkhof P. Molecular mechanisms and therapeutic targets for diabetic kidney disease. Kidney Int. (2022) 102:248–60. doi: 10.1016/j.kint.2022.05.012 35661785

[4] Zhao C Li L Li C Tang C Cai J Liu Y. PACS-2 deficiency in tubular cells aggravates lipid-related kidney injury in diabetic kidney disease. Mol Med. (2022) 28:117. doi: 10.1186/s10020-022-00545-x 36138342 PMC9502582

[5] Pereira PR Carrageta DF Oliveira PF Rodrigues A Alves MG Monteiro MP. Metabolomics as a tool for the early diagnosis and prognosis of diabetic kidney disease. Med Res Rev. (2022) 42:1518–44. doi: 10.1002/med.21883 35274315

[6] Daza-Arnedo R Rico-Fontalvo JE Pájaro-Galvis N Leal-Martínez V Abuabara-Franco E Raad-Sarabia M. Dipeptidyl peptidase-4 inhibitors and diabetic kidney disease: A narrative review. Kidney Med. (2021) 3:1065–73. doi: 10.1016/j.xkme.2021.07.007 PMC8664739 34939016

[7] DeFronzo RA Reeves WB Awad AS. Pathophysiology of diabetic kidney disease: impact of SGLT2 inhibitors. Nat Rev Nephrol. (2021) 17:319–34. doi: 10.1038/s41581-021-00393-8 33547417

[8] Kawanami D Takashi Y. GLP-1 receptor agonists in diabetic kidney disease: from clinical outcomes to mechanisms. Front Pharmacol. (2020) 11:967. doi: 10.3389/fphar.2020.00967 32694999 PMC7338581

[9] Hirano T. Pathophysiology of diabetic dyslipidemia. J Atheroscler Thromb. (2018) 25:771–82. doi: 10.5551/jat.RV17023 PMC6143775 29998913

[10] Chen X Yin Q Ma L Fu P. The role of cholesterol homeostasis in diabetic kidney disease. Curr Med Chem. (2021) 28:7413–26. doi: 10.2174/0929867328666210419132807 33874866

[11] Mitrofanova A Fontanella AM Merscher S Fornoni A. Lipid deposition and metaflammation in diabetic kidney disease. Curr Opin Pharmacol. (2020) 55:60–72. doi: 10.1016/j.coph.2020.09.004 33137677 PMC7769904

[12] Wu Y Chen Y. Research progress on ferroptosis in diabetic kidney disease. Front Endocrinol (Lausanne). (2022) 13:945976. doi: 10.3389/fendo.2022.945976 36246888 PMC9556825

[13] Feng X Wang S Sun Z Dong H Yu H Huang M. Ferroptosis Enhanced Diabetic Renal Tubular Injury via HIF-1α/HO-1 Pathway in db/db Mice. Front Endocrinol (Lausanne). (2021) 12:626390. doi: 10.3389/fendo.2021.626390 33679620 PMC7930496

[14] Yang D Livingston MJ Liu Z Dong G Zhang M Chen JK. Autophagy in diabetic kidney disease: regulation, pathological role and therapeutic potential. Cell Mol Life Sci. (2018) 75:669–88. doi: 10.1007/s00018-017-2639-1 PMC5771948 28871310

[15] Koch EAT Nakhoul R Nakhoul F Nakhoul N. Autophagy in diabetic nephropathy: a review. Int Urol Nephrol. (2020) 52:1705–12. doi: 10.1007/s11255-020-02545-4 32661628

[16] Xu YX Pu SD Li X Yu ZW Zhang YT Tong XW. Exosomal ncRNAs: Novel therapeutic target and biomarker for diabetic complications. Pharmacol Res. (2022) 178:106135. doi: 10.1016/j.phrs.2022.106135 35192956

[17] Chen X Han Y Gao P Yang M Xiao L Xiong X. Disulfide-bond A oxidoreductase-like protein protects against ectopic fat deposition and lipid-related kidney damage in diabetic nephropathy. Kidney Int. (2019) 95:880–95. doi: 10.1016/j.kint.2018.10.038 30791996

[18] Fang Q Liu N Zheng B Guo F Zeng X Huang X. Roles of gut microbial metabolites in diabetic kidney disease. Front Endocrinol (Lausanne). (2021) 12:636175. doi: 10.3389/fendo.2021.636175 34093430 PMC8173181

[19] Linh HT Iwata Y Senda Y Sakai-Takemori Y Nakade Y Oshima M. Intestinal bacterial translocation contributes to diabetic kidney disease. J Am Soc Nephrol. (2022) 33:1105–19. doi: 10.1681/ASN.2021060843 PMC9161796 35264456

[20] Chen H Liu C Wang Q Xiong M Zeng X Yang D. Renal UTX-PHGDH-serine axis regulates metabolic disorders in the kidney and liver. Nat Commun. (2022) 13:3835. doi: 10.1038/s41467-022-31476-0 35788583 PMC9253056

[21] Su K Yi B Yao BQ Xia T Yang YF Zhang ZH. Liraglutide attenuates renal tubular ectopic lipid deposition in rats with diabetic nephropathy by inhibiting lipid synthesis and promoting lipolysis. Pharmacol Res. (2020) 156:104778. doi: 10.1016/j.phrs.2020.104778 32247822

[22] Herman-Edelstein M Scherzer P Tobar A Levi M Gafter U. Altered renal lipid metabolism and renal lipid accumulation in human diabetic nephropathy. J Lipid Res. (2014) 55:561–72. doi: 10.1194/jlr.P040501 PMC3934740 24371263

[23] Yang M Han Y Luo S Xiong X Zhu X Zhao H. MAMs protect against ectopic fat deposition and lipid-related kidney damage in DN patients. Front Endocrinol (Lausanne). (2021) 12:609580. doi: 10.3389/fendo.2021.609580 33679616 PMC7933555

[24] Mitrofanova A Burke G Merscher S Fornoni A. New insights into renal lipid dysmetabolism in diabetic kidney disease. World J Diabetes. (2021) 12:524–40. doi: 10.4239/wjd.v12.i5.524 PMC8107981 33995842

[25] Tsai IT Wu CC Hung WC Lee TL Hsuan CF Wei CT. FABP1 and FABP2 as markers of diabetic nephropathy. Int J Med Sci. (2020) 17:2338–45. doi: 10.7150/ijms.49078 PMC7484639 32922199

[26] Khan S Gaivin R Abramovich C Boylan M Calles J Schelling JR. Fatty acid transport protein-2 regulates glycemic control and diabetic kidney disease progression. JCI Insight. (2020) 5. doi: 10.1172/jci.insight.136845 PMC7455077 32614804

[27] Falkevall A Mehlem A Palombo I Heller Sahlgren B Ebarasi L He L. Reducing VEGF-B signaling ameliorates renal lipotoxicity and protects against diabetic kidney disease. Cell Metab. (2017) 25:713–26. doi: 10.1016/j.cmet.2017.01.004 28190774

[28] Susztak K Ciccone E McCue P Sharma K Böttinger EP. Multiple metabolic hits converge on CD36 as novel mediator of tubular epithelial apoptosis in diabetic nephropathy. PloS Med. (2005) 2:e45. doi: 10.1371/journal.pmed.0020045 15737001 PMC549593

[29] Feng L Gu C Li Y Huang J. High glucose promotes CD36 expression by upregulating peroxisome proliferator-activated receptor γ Levels to exacerbate lipid deposition in renal tubular cells. BioMed Res Int. (2017) 2017:1414070. doi: 10.1155/2017/1414070 28497039 PMC5405368

[30] Yang X Wu Y Li Q Zhang G Wang M Yang H. CD36 promotes podocyte apoptosis by activating the pyrin domain-containing-3 (NLRP3) inflammasome in primary nephrotic syndrome. Med Sci Monit. (2018) 24:6832–9. doi: 10.12659/MSM.909810 PMC6178869 30258045

[31] Kennedy DJ Chen Y Huang W Viterna J Liu J Westfall K. CD36 and Na/K-ATPase-α1 form a proinflammatory signaling loop in kidney. Hypertension. (2013) 61:216–24. doi: 10.1161/HYPERTENSIONAHA.112.198770 PMC3521864 23172921

[32] Hua W Huang HZ Tan LT Wan JM Gui HB Zhao L. CD36 mediated fatty acid-induced podocyte apoptosis via oxidative stress. PloS One. (2015) 10:e0127507. doi: 10.1371/journal.pone.0127507 26000608 PMC4441449

[33] Kang HM Ahn SH Choi P Ko YA Han SH Chinga F. Defective fatty acid oxidation in renal tubular epithelial cells has a key role in kidney fibrosis development. Nat Med. (2015) 21:37–46. doi: 10.1038/nm.3762 25419705 PMC4444078

[34] Ito H Yamashita H Nakashima M Takaki A Yukawa C Matsumoto S. Current metabolic status affects urinary liver-type fatty-acid binding protein in normoalbuminuric patients with type 2 diabetes. J Clin Med Res. (2017) 9:366–73. doi: 10.14740/jocmr2934w PMC5330781 28270898

[35] Thi TND Gia BN Thi HLL Thi TNC Thanh HP. Evaluation of urinary L-FABP as an early marker for diabetic nephropathy in type 2 diabetic patients. J Med Biochem. (2020) 39(2):224–30. doi: 10.2478/jomb-2019-0037 PMC7526021 33033456

[36] Panduru NM Forsblom C Saraheimo M Thorn L Bierhaus A Humpert PM. Urinary liver-type fatty acid-binding protein and progression of diabetic nephropathy in type 1 diabetes. Diabetes Care. (2013) 36:2077–83. doi: 10.2337/dc12-1868 PMC3687279 23378622

[37] Viswanathan V Sivakumar S Sekar V Umapathy D Kumpatla S. Clinical significance of urinary liver-type fatty acid binding protein at various stages of nephropathy. Indian J Nephrol. (2015) 25:269–73. doi: 10.4103/0971-4065.145097 PMC4588321 26628791

[38] Ducasa GM Mitrofanova A Mallela SK Liu X Molina J Sloan A. ATP-binding cassette A1 deficiency causes cardiolipin-driven mitochondrial dysfunction in podocytes. J Clin Invest. (2019) 129:3387–400. doi: 10.1172/JCI125316 PMC6668702 31329164

[39] Han LD Xia JF Liang QL Wang Y Wang YM Hu P. Plasma esterified and non-esterified fatty acids metabolic profiling using gas chromatography-mass spectrometry and its application in the study of diabetic mellitus and diabetic nephropathy. Anal Chim Acta. (2011) 689:85–91. doi: 10.1016/j.aca.2011.01.034 21338761

[40] Zhang Y Zhang S Wang G. Metabolomic biomarkers in diabetic kidney diseases–A systematic review. J Diabetes Complications. (2015) 29:1345–51. doi: 10.1016/j.jdiacomp.2015.06.016 26253264

[41] Zhang B Wan Y Zhou X Zhang H Zhao H Ma L. Characteristics of serum metabolites and gut microbiota in diabetic kidney disease. Front Pharmacol. (2022) 13:872988. doi: 10.3389/fphar.2022.872988 35548353 PMC9084235

[42] Jiang T Liebman SE Lucia MS Li J Levi M. Role of altered renal lipid metabolism and the sterol regulatory element binding proteins in the pathogenesis of age-related renal disease. Kidney Int. (2005) 68:2608–20. doi: 10.1111/j.1523-1755.2005.00733.x 16316337

[43] Jiang T Wang Z Proctor G Moskowitz S Liebman SE Rogers T. Diet-induced obesity in C57BL/6J mice causes increased renal lipid accumulation and glomerulosclerosis via a sterol regulatory element-binding protein-1c-dependent pathway. J Biol Chem. (2005) 280:32317–25. doi: 10.1074/jbc.M500801200 16046411

[44] Proctor G Jiang T Iwahashi M Wang Z Li J Levi M. Regulation of renal fatty acid and cholesterol metabolism, inflammation, and fibrosis in Akita and OVE26 mice with type 1 diabetes. Diabetes. (2006) 55:2502–9. doi: 10.2337/db05-0603 16936198

[45] Ishigaki N Yamamoto T Shimizu Y Kobayashi K Yatoh S Sone H. Involvement of glomerular SREBP-1c in diabetic nephropathy. Biochem Biophys Res Commun. (2007) 364:502–8. doi: 10.1016/j.bbrc.2007.10.038 17961514

[46] Sun H Yuan Y Sun ZL. Cholesterol contributes to diabetic nephropathy through SCAP-SREBP-2 pathway. Int J Endocrinol. (2013) 2013:592576. doi: 10.1155/2013/592576 24369464 PMC3863482

[47] Zhou C Pridgen B King N Xu J Breslow JL. Hyperglycemic Ins2AkitaLdlr^-^/^-^ mice show severely elevated lipid levels and increased atherosclerosis: a model of type 1 diabetic macrovascular disease. J Lipid Res. (2011) 52:1483–93. doi: 10.1194/jlr.M014092 PMC3137013 21606463

[48] Woroniecka KI Park AS Mohtat D Thomas DB Pullman JM Susztak K. Transcriptome analysis of human diabetic kidney disease. Diabetes. (2011) 60:2354–69. doi: 10.2337/db10-1181 PMC3161334 21752957

[49] Ju W Greene CS Eichinger F Nair V Hodgin JB Bitzer M. Defining cell-type specificity at the transcriptional level in human disease. Genome Res. (2013) 23:1862–73. doi: 10.1101/gr.155697.113 PMC3814886 23950145

[50] Liu X Ducasa GM Mallela SK Kim JJ Molina J Mitrofanova A. Sterol-O-acyltransferase-1 has a role in kidney disease associated with diabetes and Alport syndrome. ey Int. (2020) 98:1275–85. doi: 10.1016/j.kint.2020.06.040 PMC7606642 32739420

[51] Tang C Kanter JE Bornfeldt KE Leboeuf RC Oram JF. Diabetes reduces the cholesterol exporter ABCA1 in mouse macrophages and kidneys. J Lipid Res. (2010) 51:1719–28. doi: 10.1194/jlr.M003525 PMC2882721 19965614

[52] Merscher-Gomez S Guzman J Pedigo CE Lehto M Aguillon-Prada R Mendez A. Cyclodextrin protects podocytes in diabetic kidney disease. Diabetes. (2013) 62:3817–27. doi: 10.2337/db13-0399 PMC3806621 23835338

[53] Tsun JG Yung S Chau MK Shiu SW Chan TM Tan KC. Cellular cholesterol transport proteins in diabetic nephropathy. PloS One. (2014) 9:e105787. doi: 10.1371/journal.pone.0105787 25181357 PMC4152117

[54] Sas KM Nair V Byun J Kayampilly P Zhang H Saha J. Targeted lipidomic and transcriptomic analysis identifies dysregulated renal ceramide metabolism in a mouse model of diabetic kidney disease. J Proteomics Bioinform. (2015) Suppl 14. doi: 10.4172/jpb PMC4712744 26778897

[55] Liu JJ Ghosh S Kovalik JP Ching J Choi HW Tavintharan S. Profiling of plasma metabolites suggests altered mitochondrial fuel usage and remodeling of sphingolipid metabolism in individuals with type 2 diabetes and kidney disease. Kidney Int Rep. (2017) 2:470–80. doi: 10.1016/j.ekir.2016.12.003 PMC5678636 29142974

[56] Morita Y Kurano M Sakai E Nishikawa T Nishikawa M Sawabe M. Analysis of urinary sphingolipids using liquid chromatography-tandem mass spectrometry in diabetic nephropathy. J Diabetes Investig. (2020) 11:441–9. doi: 10.1111/jdi.13154 PMC7078086 31580528

[57] Nojiri T Kurano M Tokuhara Y Ohkubo S Hara M Ikeda H. Modulation of sphingosine-1-phosphate and apolipoprotein M levels in the plasma, liver and kidneys in streptozotocin-induced diabetic mice. J Diabetes Investig. (2014) 5:639–48. doi: 10.1111/jdi.12232 PMC4234226 25422763

[58] Geoffroy K Troncy L Wiernsperger N Lagarde M El Bawab S. Glomerular proliferation during early stages of diabetic nephropathy is associated with local increase of sphingosine-1-phosphate levels. FEBS Lett. (2005) 579:1249–54. doi: 10.1016/j.febslet.2004.12.094 15710421

[59] Lan T Shen X Liu P Liu W Xu S Xie X. Berberine ameliorates renal injury in diabetic C57BL/6 mice: Involvement of suppression of SphK-S1P signaling pathway. Arch Biochem Biophys. (2010) 502:112–20. doi: 10.1016/j.abb.2010.07.012 20646989

[60] Awad AS Rouse MD Khutsishvili K Huang L Bolton WK Lynch KR. Chronic sphingosine 1-phosphate 1 receptor activation attenuates early-stage diabetic nephropathy independent of lymphocytes. Kidney Int. (2011) 79:1090–8. doi: 10.1038/ki.2010.544 PMC3155206 21289599

[61] Awad AS Ye H Huang L Li L Foss FW Jr Macdonald TL. Selective sphingosine 1-phosphate 1 receptor activation reduces ischemia-reperfusion injury in mouse kidney. Am J Physiol Renal Physiol. (2006) 290:F1516–24. doi: 10.1152/ajprenal.00311.2005 16403835

[62] Mitrofanova A Mallela SK Ducasa GM Yoo TH Rosenfeld-Gur E Zelnik ID. SMPDL3b modulates insulin receptor signaling in diabetic kidney disease. Nat Commun. (2019) 10:2692. doi: 10.1038/s41467-019-10584-4 31217420 PMC6584700

[63] Yoo TH Pedigo CE Guzman J Correa-Medina M Wei C Villarreal R. Sphingomyelinase-like phosphodiesterase 3b expression levels determine podocyte injury phenotypes in glomerular disease. J Am Soc Nephrol. (2015) 26:133–47. doi: 10.1681/ASN.2013111213 PMC4279736 24925721

[64] Ahmad A Mitrofanova A Bielawski J Yang Y Marples B Fornoni A. Sphingomyelinase-like phosphodiesterase 3b mediates radiation-induced damage of renal podocytes. FASEB J. (2017) 31(2):771–80. doi: 10.1096/fj.201600618R PMC6137603 27836988

[65] Zador IZ Deshmukh GD Kunkel R Johnson K Radin NS Shayman JA. A role for glycosphingolipid accumulation in the renal hypertrophy of streptozotocin-induced diabetes mellitus. J Clin Invest. (1993) 91:797–803. doi: 10.1172/JCI116299 8450061 PMC288030

[66] Vukovic I Bozic J Markotic A Ljubicic S Ticinovic Kurir T. The missing link - likely pathogenetic role of GM3 and other gangliosides in the development of diabetic nephropathy. Kidney Blood Press Res. (2015) 40:306–14. doi: 10.1159/000368506 26043887

[67] Hou B He P Ma P Yang X Xu C Lam SM. Comprehensive lipidome profiling of the kidney in early-stage diabetic nephropathy. Front Endocrinol (Lausanne). (2020) 11:359. doi: 10.3389/fendo.2020.00359 32655493 PMC7325916

[68] Ene CD Penescu M Anghel A Neagu M Budu V Nicolae I. Monitoring diabetic nephropathy by circulating gangliosides. J Immunoassay Immunochem. (2016) 37:68–79. doi: 10.1080/15321819.2015.1050107 26359623

[69] Du F Virtue A Wang H Yang XF. Metabolomic analyses for atherosclerosis, diabetes, and obesity. biomark Res. (2013) 1:17. doi: 10.1186/2050-7771-1-17 24252331 PMC4177614

[70] Yang Y Wang J Qin L Shou Z Zhao J Wang H. Rapamycin prevents early steps of the development of diabetic nephropathy in rats. Am J Nephrol. (2007) 27:495–502. doi: 10.1159/000106782 17671379

[71] Zhao YY. Metabolomics in chronic kidney disease. Clin Chim Acta. (2013) 422:59–69. doi: 10.1016/j.cca.2013.03.033 23570820

[72] Zhao YY Vaziri ND Lin RC. Lipidomics: new insight into kidney disease. Adv Clin Chem. (2015) 68:153–75. doi: 10.1016/bs.acc.2014.11.002 25858872

[73] Jiang Z Liang Q Luo G Hu P Li P Wang Y. HPLC-electrospray tandem mass spectrometry for simultaneous quantitation of eight plasma aminothiols: application to studies of diabetic nephropathy. Talanta. (2009) 77:1279–84. doi: 10.1016/j.talanta.2008.08.031 19084635

[74] Karpe F Dickmann JR Frayn KN. Fatty acids, obesity, and insulin resistance: time for a reevaluation. Diabetes. (2011) 60:2441–9. doi: 10.2337/db11-0425 PMC3178283 21948998

[75] Smith CA O’Maille G Want EJ Qin C Trauger SA Brandon TR. METLIN - a metabolite mass spectral database. Ther Drug Monit. (2005) 27:747–51. doi: 10.1097/01.ftd.0000179845.53213.39 16404815

[76] Fahy E Sud M Cotter D Subramaniam S. LIPID MAPS online tools for lipid research. Nucleic Acids Res. (2007) 35:W606–12. doi: 10.1093/nar/gkm324 PMC1933166 17584797

[77] Grove KJ Voziyan PA Spraggins JM Wang S Paueksakon P Harris RC. Diabetic nephropathy induces alterations in the glomerular and tubule lipid profiles. J Lipid Res. (2014) 55:1375–85. doi: 10.1194/jlr.M049189 PMC4076088 24864273

[78] Zhu C Liang QL Hu P Wang YM Luo GA. Phospholipidomic identification of potential plasma biomarkers associated with type 2 diabetes mellitus and diabetic nephropathy. Talanta. (2011) 85:1711–20. doi: 10.1016/j.talanta.2011.05.036 21872008

[79] Zhang G Zhang J DeHoog RJ Pennathur S Anderton CR Venkatachalam MA. DESI-MSI and METASPACE indicates lipid abnormalities and altered mitochondrial membrane components in diabetic renal proximal tubules. Metabolomics. (2020) 16:11. doi: 10.1007/s11306-020-1637-8 31925564 PMC7301343

[80] Melo RC D’Avila H Wan HC Bozza PT Dvorak AM Weller PF. Lipid bodies in inflammatory cells: structure, function, and current imaging techniques. J Histochem Cytochem. (2011) 59:540–56. doi: 10.1369/0022155411404073 PMC3201176 21430261

[81] Urahama Y Ohsaki Y Fujita Y Maruyama S Yuzawa Y Matsuo S. Lipid droplet-associated proteins protect renal tubular cells from fatty acid-induced apoptosis. Am J Pathol. (2008) 173:1286–94. doi: 10.2353/ajpath.2008.080137 PMC2570120 18832575

[82] Garbarino J Padamsee M Wilcox L Oelkers PM D’ AD Ruggles K. Sterol and diacylglycerol acyltransferase deficiency triggers fatty acid mediated cell death. J Biol Chem. (2009) 284:30994–1005. doi: 10.1074/jbc.M109.050443 PMC2781500 19690167

[83] Greenberg AS Coleman RA Kraemer FB McManaman JL Obin MS Puri V. The role of lipid droplets in metabolic disease in rodents and humans. J Clin Invest. (2011) 121:2102–10. doi: 10.1172/JCI46069 PMC3104768 21633178

[84] Olzmann JA Carvalho P. Dynamics and functions of lipid droplets. Nat Rev Mol Cell Biol. (2019) 20:137–55. doi: 10.1038/s41580-018-0085-z PMC6746329 30523332

[85] Wang Z Jiang T Li J Proctor G McManaman JL Lucia S. Regulation of renal lipid metabolism, lipid accumulation, and glomerulosclerosis in FVBdb/db mice with type 2 diabetes. Diabetes. (2005) 54:2328–35. doi: 10.2337/diabetes.54.8.2328 16046298

[86] Kiss E Kränzlin B Wagenblaβ K Bonrouhi M Thiery J Gröne E. Lipid droplet accumulation is associated with an increase in hyperglycemia-induced renal damage: prevention by liver X receptors. Am J Pathol. (2013) 182:727–41. doi: 10.1016/j.ajpath.2012.11.033 23318573

[87] Yang W Luo Y Yang S Zeng M Zhang S Liu J. Ectopic lipid accumulation: potential role in tubular injury and inflammation in diabetic kidney disease. Clin Sci (Lond). (2018) 132:2407–22. doi: 10.1042/CS20180702 30348828

[88] Wang X Chen C Xie C Huang W Young RL Jones KL. Serum bile acid response to oral glucose is attenuated in patients with early type 2 diabetes and correlates with 2-hour plasma glucose in individuals without diabetes. Diabetes Obes Metab. (2022) 24:1132–42. doi: 10.1111/dom.14683 PMC9540586 35238131

[89] Xiao X Zhang J Ji S Qin C Wu Y Zou Y. Lower bile acids as an independent risk factor for renal outcomes in patients with type 2 diabetes mellitus and biopsy-proven diabetic kidney disease. Front Endocrinol (Lausanne). (2022) 13:1026995. doi: 10.3389/fendo.2022.1026995 36277729 PMC9585231

[90] Jiang T Wang XX Scherzer P Wilson P Tallman J Takahashi H. Farnesoid X receptor modulates renal lipid metabolism, fibrosis, and diabetic nephropathy. Diabetes. (2007) 56:2485–93. doi: 10.2337/db06-1642 17660268

[91] Arsenijevic D Cajot JF Fellay B Dulloo AG Van Vliet BN Montani JP. Uninephrectomy-induced lipolysis and low-grade inflammation are mimicked by unilateral renal denervation. Front Physiol. (2016) 7:227. doi: 10.3389/fphys.2016.00227 27378937 PMC4906570

[92] Xiong F Li X Yang Z Wang Y Gong W Huang J. TGR5 suppresses high glucose-induced upregulation of fibronectin and transforming growth factor-β1 in rat glomerular mesangial cells by inhibiting RhoA/ROCK signaling. Endocrine. (2016) 54:657–70. doi: 10.1007/s12020-016-1032-4 27470217

[93] Yang Z Xiong F Wang Y Gong W Huang J Chen C. TGR5 activation suppressed S1P/S1P2 signaling and resisted high glucose-induced fibrosis in glomerular mesangial cells. Pharmacol Res. (2016) 111:226–36. doi: 10.1016/j.phrs.2016.05.035 27317945

[94] Wang XX Edelstein MH Gafter U Qiu L Luo Y Dobrinskikh E. G Protein-coupled bile acid receptor TGR5 activation inhibits kidney disease in obesity and diabetes. J Am Soc Nephrol. (2016) 27:1362–78. doi: 10.1681/ASN.2014121271 PMC4849814 26424786

[95] Podrini C Rowe I Pagliarini R Costa ASH Chiaravalli M Di Meo I. Dissection of metabolic reprogramming in polycystic kidney disease reveals coordinated rewiring of bioenergetic pathways. Commun Biol. (2018) 1:194. doi: 10.1038/s42003-018-0200-x 30480096 PMC6240072

[96] Cargill K Sims-Lucas S. Metabolic requirements of the nephron. Pediatr Nephrol. (2020) 35:1–8. doi: 10.1007/s00467-018-4157-2 30554363

[97] Njeim R Alkhansa S Fornoni A. Unraveling the crosstalk between lipids and NADPH oxidases in diabetic kidney disease. Pharmaceutics. (2023) 15:1360. doi: 10.3390/pharmaceutics15051360 37242602 PMC10222020

[98] Brown MS Goldstein JL. The SREBP pathway: regulation of cholesterol metabolism by proteolysis of a membrane-bound transcription factor. Cell. (1997) 89:331–40. doi: 10.1016/s0092-8674(00)80213-5 9150132

[99] Hao J Zhu L Zhao S Liu S Liu Q Duan H. PTEN ameliorates high glucose-induced lipid deposits through regulating SREBP-1/FASN/ACC pathway in renal proximal tubular cells. Exp Cell Res. (2011) 317:1629–39. doi: 10.1016/j.yexcr.2011.02.003 21320485

[100] Sun L Halaihel N Zhang W Rogers T Levi M. Role of sterol regulatory element-binding protein 1 in regulation of renal lipid metabolism and glomerulosclerosis in diabetes mellitus. J Biol Chem. (2002) 277:18919–27. doi: 10.1074/jbc.M110650200 11875060

[101] Wang H Zhu L Hao J Duan H Liu S Zhao S. Co-regulation of SREBP-1 and mTOR ameliorates lipid accumulation in kidney of diabetic mice. Exp Cell Res. (2015) 336(1):76–84. doi: 10.1016/j.yexcr.2015.06.006 26112216

[102] Szolkiewicz M Chmielewski M Nogalska A Stelmanska E Swierczynski J Rutkowski B. The potential role of sterol regulatory element binding protein transcription factors in renal injury. J Ren Nutr. (2007) 17:62–5. doi: 10.1053/j.jrn.2006.10.009 17198935

[103] Jun H Song Z Chen W Zanhua R Yonghong S Shuxia L. *In vivo* and in *vitro* effects of SREBP-1 on diabetic renal tubular lipid accumulation and RNAi-mediated gene silencing study. Histochem Cell Biol. (2009) 131(3):327–45. doi: 10.1007/s00418-008-0528-2 19048273

[104] Nosadini R Tonolo G. Role of oxidized low density lipoproteins and free fatty acids in the pathogenesis of glomerulopathy and tubulointerstitial lesions in type 2 diabetes. Nutr Metab Cardiovasc Dis. (2011) 21:79–85. doi: 10.1016/j.numecd.2010.10.002 21186102

[105] Lee HS Lee SK. Intraglomerular lipid deposition in renal disease. Miner Electrolyte Metab. (1993) 19:144–8. 8232101

[106] Ruan XZ Varghese Z Powis SH Moorhead JF. Dysregulation of LDL receptor under the influence of inflammatory cytokines: a new pathway for foam cell formation. Kidney Int. (2001) 60:1716–25. doi: 10.1046/j.1523-1755.2001.00025.x 11703589

[107] Attie AD. ABCA1: at the nexus of cholesterol, HDL and atherosclerosis. Trends Biochem Sci. (2007) 32:172–9. doi: 10.1016/j.tibs.2007.02.001 17324574

[108] Shiffman D Pare G Oberbauer R Louie JZ Rowland CM Devlin JJ. A gene variant in CERS2 is associated with rate of increase in albuminuria in patients with diabetes from ONTARGET and TRANSCEND. PloS One. (2014) 9(9):e106631. doi: 10.1371/journal.pone.0106631 25238615 PMC4169514

[109] Lovric S Goncalves S Gee HY Oskouian B Srinivas H Choi WI. Mutations in sphingosine-1-phosphate lyase cause nephrosis with ichthyosis and adrenal insufficiency. J Clin Invest. (2017) 127(3):912–28. doi: 10.1172/JCI89626 PMC5330730 28165339

[110] Mitrofanova A Sosa MA Fornoni A. Lipid mediators of insulin signaling in diabetic kidney disease. Am J Physiol Renal Physiol. (2019) 317:F1241–52. doi: 10.1152/ajprenal.00379.2019 PMC6879940 31545927

[111] Chen RX Zhang L Ye W Wen YB Si N Li H. The renal manifestations of type 4 familial partial lipodystrophy: a case report and review of literature. BMC Nephrol. (2018) 19(1):111. doi: 10.1186/s12882-018-0913-6 29747582 PMC5946515

[112] Saravani R Noorzehi N Galavi HR Ranjbar N Lotfian Sargazi M. Association of perilipin and insulin receptor substrate-1 genes polymorphism with lipid profiles, central obesity, and type 2 diabetes in a sample of an Iranian population. Iran Red Crescent Med J. (2017) 19:e55100. doi: 10.5812/ircmj

[113] Kullak-Ublick GA Hagenbuch B Stieger B Schteingart CD Hofmann AF Wolkoff AW. Molecular and functional characterization of an organic anion transporting polypeptide cloned from human liver. Gastroenterology. (1995) 109(4):1274–82. doi: 10.1016/0016-5085(95)90588-X 7557095

[114] More VR Wen X Thomas PE Aleksunes LM Slitt AL. Severe diabetes and leptin resistance cause differential hepatic and renal transporter expression in mice. Comp Hepatol. (2012) 11:1. doi: 10.1186/1476-5926-11-1 22524730 PMC3416584

[115] Zhang H Zhao T Li Z Yan M Zhao H Zhu B. Transcriptional profile of kidney from type 2 diabetic db/db mice. J Diabetes Res. (2017) 2017:8391253. doi: 10.1155/2017/8391253 28232950 PMC5292381

[116] Atlas D. International diabetes federation[J]. IDF Diabetes Atlas. 7th edn. Brussels, Belgium: International Diabetes Federation (2015) 33(2).

[117] Sharma SK Panneerselvam A Singh KP Parmar G Gadge P Swami OC. Teneligliptin in management of type 2 diabetes mellitus. Diabetes Metab Syndr Obes. (2016) 9:251–60. doi: 10.2147/DMSO PMC4993264 27574456

[118] Cansby E Caputo M Gao L Kulkarni NM Nerstedt A Ståhlman M. Depletion of protein kinase STK25 ameliorates renal lipotoxicity and protects against diabetic kidney disease. JCI Insight. (2020) 5:e140483. doi: 10.1172/jci.insight.140483 33170807 PMC7819747

[119] Gonzalez CD Carro Negueruela MP Nicora Santamarina C Resnik R Vaccaro MI. Autophagy dysregulation in diabetic kidney disease: from pathophysiology to pharmacological interventions. Cells. (2021) 10:2497. doi: 10.3390/cells10092497 34572148 PMC8469825

[120] Ao L Xie Y. Research advance in the mechanism for oxidative stress-induced podocyte injury in diabetic kidney disease. Zhong Nan Da Xue Xue Bao Yi Xue Ban. (2021) 46:1403–8. doi: 10.11817/j.issn.1672-7347.2021.210199 PMC10930572 35232911

[121] Perkovic V Jardine MJ Neal B Bompoint S Heerspink HJL Charytan DM. Canagliflozin and renal outcomes in type 2 diabetes and nephropathy. N Engl J Med. (2019) 380(24):2295–306. doi: 10.1056/NEJMoa1811744 30990260

[122] Abubaker M Mishra P Swami OC. Teneligliptin in management of diabetic kidney disease: A review of place in therapy. J Clin Diagn Res. (2017) 11:OE05–9. doi: 10.7860/JCDR/2017/25060.9228 PMC5324442 28273997

[123] Pavkov ME Collins AJ Coresh J Nelson RG. Kidney disease in diabetes. In: Cowie CC Casagrande SS Menke A, editors. Diabetes in America. 3rd edn. Bethesda, MD: National Institute of Diabetes and Digestive and Kidney Diseases (US). (2018).

[124] American Diabetes Association. Microvascular complications and foot care: standards of medical care in diabetes. Diabetes Care. (2020) 43:S135–51. doi: 10.2337/dc20-S011 31862754

[125] Lu Q Yang L Xiao JJ Liu Q Ni L Hu JW. Empagliflozin attenuates the renal tubular ferroptosis in diabetic kidney disease through AMPK/NRF2 pathway. Free Radic Biol Med. (2023) 195:89–102. doi: 10.1016/j.freeradbiomed.2022.12.088 36581059

[126] Wu X Li H Wan Z Wang R Liu J Liu Q. The combination of ursolic acid and empagliflozin relieves diabetic nephropathy by reducing inflammation, oxidative stress and renal fibrosis. BioMed Pharmacother. (2021) 144:112267. doi: 10.1016/j.biopha.2021.112267 34624679

[127] Li D Li Y. The interaction between ferroptosis and lipid metabolism in cancer. Signal Transduct Target Ther. (2020) 5:108. doi: 10.1038/s41392-020-00216-5 32606298 PMC7327075

[128] Bao WD Pang P Zhou XT Hu F Xiong W Chen K. Loss of ferroportin induces memory impairment by promoting ferroptosis in Alzheimer’s disease. Cell Death Differ. (2021) 28:1548–62. doi: 10.1038/s41418-020-00685-9 PMC8166828 33398092

[129] Fang X Ardehali H Min J Wang F. The molecular and metabolic landscape of iron and ferroptosis in cardiovascular disease. Nat Rev Cardiol. (2023) 20:7–23. doi: 10.1038/s41569-022-00735-4 35788564 PMC9252571

[130] Wang J Liu Y Wang Y Sun L. The cross-link between ferroptosis and kidney diseases. Oxid Med Cell Longev. (2021) 2021:6654887. doi: 10.1155/2021/6654887 34007403 PMC8110383

[131] Adiyanti SS Loho T. Acute kidney injury (AKI) biomarker. Acta Med Indones. (2012) 44:246–55. 22983082

[132] Akatsuka S Yamashita Y Ohara H Liu YT Izumiya M Abe K. Fenton reaction induced cancer in wild type rats recapitulates genomic alterations observed in human cancer. PloS One. (2012) 7:e43403. doi: 10.1371/journal.pone.0043403 22952676 PMC3430702

[133] Kim JW Lee JY Oh M Lee EW. An integrated view of lipid metabolism in ferroptosis revisited via lipidomic analysis. Exp Mol Med. (2023) 55:1620–31. doi: 10.1038/s12276-023-01077-y PMC10474074 37612411

[134] Zielinski ZAM Pratt DA. Cholesterol autoxidation revisited: debunking the dogma associated with the most vilified of lipids. J Am Chem Soc. (2016) 138:6932–5. doi: 10.1021/jacs.6b03344 27210001

[135] Kitada M Koya D. Autophagy in metabolic disease and ageing. Nat Rev Endocrinol. (2021) 17:647–61. doi: 10.1038/s41574-021-00551-9 34508250

[136] Deretic V. Autophagy in inflammation, infection, and immunometabolism. Immunity. (2021) 54:437–53. doi: 10.1016/j.immuni.2021.01.018 PMC8026106 33691134

[137] Yu J Liu Y Li H Zhang P. Pathophysiology of diabetic kidney disease and autophagy: A review. Med (Baltimore). (2023) 102:e33965. doi: 10.1097/MD.0000000000033965 PMC10378892 37505163

[138] Zhang Z Sun Y Xue J Jin D Li X Zhao D. The critical role of dysregulated autophagy in the progression of diabetic kidney disease. Front Pharmacol. (2022) 13:977410. doi: 10.3389/fphar.2022.977410 36091814 PMC9453227

[139] Liu P Zhu W Wang Y Ma G Zhao H Li P. Chinese herbal medicine and its active compounds in attenuating renal injury via regulating autophagy in diabetic kidney disease. Front Endocrinol (Lausanne). (2023) 14:1142805. doi: 10.3389/fendo.2023.1142805 36942026 PMC10023817

[140] Li Z Nakatogawa H. Degradation of nuclear components via different autophagy pathways. Trends Cell Biol. (2022) 32:574–84. doi: 10.1016/j.tcb.2021.12.008 35067425

[141] Choi AM Ryter SW Levine B. Autophagy in human health and disease. N Engl J Med. (2013) 368:1845–6. doi: 10.1056/NEJMc1303158 23656658

[142] Dikic I Elazar Z. Mechanism and medical implications of mammalian autophagy. Nat Rev Mol Cell Biol. (2018) 19:349–64. doi: 10.1038/s41580-018-0003-4 29618831

[143] Singh R Kaushik S Wang Y Xiang Y Novak I Komatsu M. Autophagy regulates lipid metabolism. Nature. (2009) 458:1131–5. doi: 10.1038/nature07976 PMC2676208 19339967

[144] Zhao X Amevor FK Cui Z Wan Y Xue X Peng C. Corrigendum to “Steatosis in metabolic diseases: A focus on lipolysis and lipophagy”. BioMed Pharmacother. (2023) 163:114842. doi: 10.1016/j.biopha.2023.114842 37150633

[145] Lan ZQ Ge ZY Lv SK Zhao B Li CX. The regulatory role of lipophagy in central nervous system diseases. Cell Death Discovery. (2023) 9:229. doi: 10.1038/s41420-023-01504-z 37414782 PMC10326259

[146] Shin DW. Lipophagy: molecular mechanisms and implications in metabolic disorders. Mol Cells. (2020) 43:686–93. doi: 10.14348/molcells.2020.0046 PMC7468585 32624503

[147] Zhang X Evans TD Jeong SJ Razani B. Classical and alternative roles for autophagy in lipid metabolism. Curr Opin Lipidol. (2018) 29:203–11. doi: 10.1097/MOL.0000000000000509 PMC5930069 29601311

[148] Schelling JR. The contribution of lipotoxicity to diabetic kidney disease. Cells. (2022) 11:3236. doi: 10.3390/cells11203236 36291104 PMC9601125

[149] Yan Q Song Y Zhang L Chen Z Yang C Liu S. Autophagy activation contributes to lipid accumulation in tubular epithelial cells during kidney fibrosis [published correction appears in Cell Death Discov. 2019 Jul 10;5:116]. Cell Death Discovery. (2018) 4:2. doi: 10.1038/s41420-018-0065-2 PMC6060103 30062051

[150] Sathyanarayan A Mashek MT Mashek DG. ATGL promotes autophagy/lipophagy via SIRT1 to control hepatic lipid droplet catabolism. Cell Rep. (2017) 19:1–9. doi: 10.1016/j.celrep.2017.03.026 28380348 PMC5396179

[151] Yacoub R Lee K He JC. The role of SIRT1 in diabetic kidney disease. Front Endocrinol (Lausanne). (2014) 5:166. doi: 10.3389/fendo.2014.00166 25346724 PMC4191277

[152] Ji J Tao P Wang Q Li L Xu Y. SIRT1: mechanism and protective effect in diabetic nephropathy. Endocr Metab Immune Disord Drug Targets. (2021) 21:835–42. doi: 10.2174/1871530320666201029143606 33121427

[153] Yang L Li P Fu S Calay ES Hotamisligil GS. Defective hepatic autophagy in obesity promotes ER stress and causes insulin resistance. Cell Metab. (2010) 11:467–78. doi: 10.1016/j.cmet.2010.04.005 PMC2881480 20519119

[154] Pressly JD Gurumani MZ Varona Santos JT Fornoni A Merscher S Al-Ali H. Adaptive and maladaptive roles of lipid droplets in health and disease. Am J Physiol Cell Physiol. (2022) 322:C468–81. doi: 10.1152/ajpcell.00239.2021 PMC8917915 35108119

[155] Zhang H Yan S Khambu B Ma F Li Y Chen X. Dynamic MTORC1-TFEB feedback signaling regulates hepatic autophagy, steatosis and liver injury in long-term nutrient oversupply. Autophagy. (2018) 14(10):1779–95. doi: 10.1080/15548627.2018.1490850 PMC6135624 30044707

[156] Seo AY Lau PW Feliciano D Sengupta P Gros MAL Cinquin B. AMPK and vacuole-associated Atg14p orchestrate μ-lipophagy for energy production and long-term survival under glucose starvation. Elife. (2017) 6:e21690. doi: 10.7554/eLife.21690 28394250 PMC5407857

[157] Brosius FC Coward RJ. Podocytes, signaling pathways, and vascular factors in diabetic kidney disease. Adv Chronic Kidney Dis. (2014) 21:304–10. doi: 10.1053/j.ackd.2014.03.011 PMC4075065 24780459

[158] Wang Y Zhao H Wang Q Zhou X Lu X Liu T. Chinese herbal medicine in ameliorating diabetic kidney disease via activating autophagy. J Diabetes Res. (2019) 2019:9030893. doi: 10.1155/2019/9030893 31828168 PMC6885296

[159] Yang Z Klionsky DJ. Eaten alive: a history of macroautophagy. Nat Cell Biol. (2010) 12:814–22. doi: 10.1038/ncb0910-814 PMC3616322 20811353

[160] Yamagata M Obara K Kihara A. Sphingolipid synthesis is involved in autophagy in Saccharomyces cerevisiae. Biochem Biophys Res Commun. (2011) 410:786–91. doi: 10.1016/j.bbrc.2011.06.061 21703229

[161] Drexler Y Molina J Mitrofanova A Fornoni A Merscher S. Sphingosine-1-phosphate metabolism and signaling in kidney diseases. J Am Soc Nephrol. (2021) 32:9–31. doi: 10.1681/ASN.2020050697 33376112 PMC7894665

[162] Scarlatti F Bauvy C Ventruti A Sala G Cluzeaud F Vandewalle A. Ceramide-mediated macroautophagy involves inhibition of protein kinase B and up-regulation of beclin 1. J Biol Chem. (2004) 279(18):18384–91. doi: 10.1074/jbc.M313561200 14970205

[163] Kim SJ Tang T Abbott M Viscarra JA Wang Y Sul HS. AMPK phosphorylates desnutrin/ATGL and hormone-sensitive lipase to regulate lipolysis and fatty acid oxidation within adipose tissue. Mol Cell Biol. (2016) 36:1961–76. doi: 10.1128/MCB.00244-16 PMC4936063 27185873

[164] Dodson M Darley-Usmar V Zhang J. Cellular metabolic and autophagic pathways: traffic control by redox signaling. Free Radic Biol Med. (2013) 63:207–21. doi: 10.1016/j.freeradbiomed.2013.05.014 PMC3729625 23702245

[165] Ng MYW Charsou C Lapao A Singh S Trachsel-Moncho L Schultz SW. The cholesterol transport protein GRAMD1C regulates autophagy initiation and mitochondrial bioenergetics. Nat Commun. (2022) 13(1):6283. doi: 10.1038/s41467-022-33933-2 36270994 PMC9586981

[166] Lin Z Long F Kang R Klionsky DJ Yang M Tang D. The lipid basis of cell death and autophagy. Autophagy. (2024) 20(3):469–88. doi: 10.1080/15548627.2023.2259732 PMC10936693 37768124

[167] Yang C Zhang Z Liu J Chen P Li J Shu H. Research progress on multiple cell death pathways of podocytes in diabetic kidney disease. Mol Med. (2023) 29(1):135. doi: 10.1186/s10020-023-00732-4 37828444 PMC10571269

[168] Singh A Mittal M. Neonatal microbiome-a brief review. J Matern Fetal Neona. (2020) 33:3841–8. doi: 10.1080/14767058.2019.1583738 30835585

[169] McLean MH Dieguez D Jr Miller LM Young HA. Does the microbiota play a role in the pathogenesis of autoimmune diseases? Gut. (2015) 64:332–41. doi: 10.1136/gutjnl-2014-308514 PMC6288812 25416067

[170] Wu HJ Wu E. The role of gut microbiota in immune homeostasis and autoimmunity. Gut Microbes. (2012) 3:4–14. doi: 10.4161/gmic.19320 22356853 PMC3337124

[171] Sanidad KZ Amir M Ananthanarayanan A Singaraju A Shiland NB Hong HS. Maternal gut microbiome-induced IgG regulates neonatal gut microbiome and immunity. Sci Immunol. (2022) 7(72):eabh3816. doi: 10.1126/sciimmunol.abh3816 35687695 PMC9375732

[172] Brown EM Kenny DJ Xavier RJ. Gut microbiota regulation of t cells during inflammation and autoimmunity. Annu Rev Immunol. (2019) 37:599–624. doi: 10.1146/annurev-immunol-042718-041841 31026411

[173] Rooks MG Garrett WS. Gut microbiota, metabolites and host immunity. Nat Rev Immunol. (2016) 16:341–52. doi: 10.1038/nri.2016.42 PMC5541232 27231050

[174] Chang PV Hao LM Offermanns S Medzhitov R. The microbial metabolite butyrate regulates intestinal macrophage function via histone deacetylase inhibition. Proc Natl Acad Sci USA. (2014) 111(6):2247–52. doi: 10.1073/pnas.1322269111 PMC3926023 24390544

[175] Tian Y Gui W Koo I Smith PB Allman EL Nichols RG. The microbiome modulating activity of bile acids. Gut Microbes. (2020) 11(4):979–96. doi: 10.1080/19490976.2020.1732268 PMC7524280 32138583

[176] Lanza M Filippone A Ardizzone A Casili G Paterniti I Esposito E. SCFA treatment alleviates pathological signs of migraine and related intestinal alterations in a mouse model of NTG-induced migraine. Cells. (2021) 10:2756. doi: 10.3390/cells10102756 34685736 PMC8535085

[177] McGlone ER Bloom SR. Bile acids and the metabolic syndrome. Ann Clin Biochem. (2019) 56:326–37. doi: 10.1177/0004563218817798 30453753

[178] Shao JW Ge TT Chen SZ Wang G Yang Q Huang CH. Role of bile acids in liver diseases mediated by the gut microbiome. World J Gastroenterol. (2021) 27(22):3010–21. doi: 10.3748/wjg.v27.i22.3010 PMC8192287 34168404

[179] Chi MX Ma K Wang J Ding Z Li Y Zhu S. The immunomodulatory effect of the gut microbiota in kidney disease. J Immunol Res. (2021) 15:5516035. doi: 10.1155/2021/5516035 PMC8140847 34095319

[180] Zaky A Glastras SJ Wong MYW Pollock CA Saad S. The role of the gut microbiome in diabetes and obesity-related kidney disease. Int J Mol Sci. (2021) 22:9641. doi: 10.3390/ijms22179641 34502562 PMC8431784

[181] Amini Khiabani S Asgharzadeh M Samadi Kafil H. Chronic kidney disease and gut microbiota. Heliyon. (2023) 9:e18991. doi: 10.1016/j.heliyon.2023.e18991 37609403 PMC10440536

[182] Bush KT Wu W Lun C Nigam SK. The drug transporter OAT3 (SLC22A8) and endogenous metabolite communication via the gut-liver-kidney axis. J Biol Chem. (2017) 292:15789–803. doi: 10.1074/jbc.M117.796516 PMC5612110 28765282

[183] Tan JK McKenzie C Mariño E Macia L Mackay CR. Metabolite-sensing G protein-coupled receptors-facilitators of diet-related immune regulation. Annu Rev Immunol. (2017) 35:371–402. doi: 10.1146/annurev-immunol-051116-052235 28446062

[184] Thorburn AN Macia L Mackay CR. Diet, metabolites, and “Western-lifestyle” inflammatory diseases. Immunity. (2014) 40:833–42. doi: 10.1016/j.immuni.2014.05.014 24950203

[185] Liao D Liu YQ Xiong LY Zhang L. Renoprotective effect of atorvastatin on STZ-diabetic rats through inhibiting inflammatory factors expression in diabetic rat. Eur Rev Med Pharmacol Sci. (2016) 20:1888–93. 27212184

[186] Tang C Deng X Qu J Miao Y Tian L Zhang M. Fenofibrate attenuates renal tubular cell apoptosis by up-regulating MCAD in diabetic kidney disease. Drug Des Devel Ther. (2023) 17:1503–14. doi: 10.2147/DDDT.S405266 PMC10202114 37223723

[187] Wang S Yang Z Xiong F Chen C Chao X Huang J. Betulinic acid ameliorates experimental diabetic-induced renal inflammation and fibrosis via inhibiting the activation of NF-κB signaling pathway. Mol Cell Endocrinol. (2016) 434:135–43. doi: 10.1016/j.mce.2016.06.019 27364889

[188] Yamada S Tanabe J Ogura Y Nagai Y Sugaya T Ohata K. Renoprotective effect of GLP-1 receptor agonist, liraglutide, in early-phase diabetic kidney disease in spontaneously diabetic Torii fatty rats. Clin Exp Nephrol. (2021) 25:365–75. doi: 10.1007/s10157-020-02007-2 33409761

[189] Dugbartey GJ Alornyo KK Diaba DE Adams I. Activation of renal CSE/H2S pathway by alpha-lipoic acid protects against histological and functional changes in the diabetic kidney. BioMed Pharmacother. (2022) 153:113386. doi: 10.1016/j.biopha.2022.113386 35834985

[190] Choi SR Lim JH Kim MY Kim EN Kim Y Choi BS. Adiponectin receptor agonist AdipoRon decreased ceramide, and lipotoxicity, and ameliorated diabetic nephropathy. Metabolism. (2018) 85:348–60. doi: 10.1016/j.metabol.2018.02.004 29462574

[191] Kim Y Lim JH Kim MY Kim EN Yoon HE Shin SJ. The adiponectin receptor agonist adipoRon ameliorates diabetic nephropathy in a model of type 2 diabetes. J Am Soc Nephrol. (2018) 29:1108–27. doi: 10.1681/ASN.2017060627 PMC5875945 29330340

[192] Santana MFM Lira ALA Pinto RS Minanni CA Silva ARM Sawada MIBAC. Enrichment of apolipoprotein A-IV and apolipoprotein D in the HDL proteome is associated with HDL functions in diabetic kidney disease without dialysis. Lipids Health Dis. (2020) 19:205. doi: 10.1186/s12944-020-01381-w 32921312 PMC7488728

[193] Zhou Y Liu L Jin B Wu Y Xu L Chang X. Metrnl alleviates lipid accumulation by modulating mitochondrial homeostasis in diabetic nephropathy. Diabetes. (2023) 72:611–26. doi: 10.2337/db22-0680 PMC10130489 36812572

[194] Lin S Wang L Jia Y Sun Y Qiao P Quan Y. Lipin-1 deficiency deteriorates defect of fatty acid β-oxidation and lipid-related kidney damage in diabetic kidney disease. Transl Res. (2023) 266:1–15. doi: 10.1016/j.trsl.2023.07.004 37433392

[195] Liu S Da J Yu J Dong R Yuan J Yu F. Renal tubule ectopic lipid deposition in diabetic kidney disease rat model and in *vitro* mechanism of leptin intervention. J Physiol Biochem. (2022) 78:389–99. doi: 10.1007/s13105-022-00874-9 35192189

[196] Zhang J Wu Y Zhang J Zhang R Wang Y Liu F. ABCA1 deficiency-mediated glomerular cholesterol accumulation exacerbates glomerular endothelial injury and dysfunction in diabetic kidney disease. Metabolism. (2023) 139:155377. doi: 10.1016/j.metabol.2022.155377 36521550

[197] Li X Xu B Wu J Pu Y Wan S Zeng Y. Maresin 1 Alleviates Diabetic Kidney Disease via LGR6-Mediated cAMP-SOD2-ROS Pathway. Oxid Med Cell Longev. (2022) 2022:7177889. doi: 10.1155/2022/7177889 35498124 PMC9042615

[198] Yu M Wang D Zhong D Xie W Luo J. Adropin carried by reactive oxygen species-responsive nanocapsules ameliorates renal lipid toxicity in diabetic mice. ACS Appl Mater Interfaces. (2022) 14:37330–44. doi: 10.1021/acsami.2c06957 35951354

[199] Castañeda TR Méndez M Davison I Elvert R Schwahn U Boldina G. The novel phosphate and bile acid sequestrant polymer SAR442357 delays disease progression in a rat model of diabetic nephropathy. J Pharmacol Exp Ther. (2021) 376:190–203. doi: 10.1124/jpet.120.000285 33203659

[200] Sun ZJ Chang DY Chen M Zhao MH. Deficiency of CFB attenuates renal tubulointerstitial damage by inhibiting ceramide synthesis in diabetic kidney disease. JCI Insight. (2022) 7:e156748. doi: 10.1172/jci.insight.156748 36546481 PMC9869976

[201] Qin X Jiang M Zhao Y Gong J Su H Yuan F. Berberine protects against diabetic kidney disease via promoting PGC-1α-regulated mitochondrial energy homeostasis. Br J Pharmacol. (2020) 177:3646–61. doi: 10.1111/bph.14935 PMC7393204 31734944

[202] Qin X Zhao Y Gong J Huang W Su H Yuan F. Berberine protects glomerular podocytes via inhibiting drp1-mediated mitochondrial fission and dysfunction. Theranostics. (2019) 9:1698–713. doi: 10.7150/thno.30640 PMC6485199 31037132

[203] Wang FL Tang LQ Yang F Zhu LN Cai M Wei W. Renoprotective effects of berberine and its possible molecular mechanisms in combination of high-fat diet and low-dose streptozotocin-induced diabetic rats. Mol Biol Rep. (2013) 40:2405–18. doi: 10.1007/s11033-012-2321-5 23196710

[204] Wu R Liang Y Xu M Fu K Zhang Y Wu L. Advances in chemical constituents, clinical applications, pharmacology, pharmacokinetics and toxicology of erigeron breviscapus. Front Pharmacol. (2021) 12:656335. doi: 10.3389/fphar.2021.656335 34539390 PMC8443777

[205] Liu X Yao L Sun D Zhu X Liu Q Xu T. Effect of breviscapine injection on clinical parameters in diabetic nephropathy: A meta-analysis of randomized controlled trials. Exp Ther Med. (2016) 12:1383–97. doi: 10.3892/etm.2016.3483 PMC4998064 27588060

[206] Udrea AM Gradisteanu Pircalabioru G Boboc AA Mares C Dinache A Mernea M. Advanced bioinformatics tools in the pharmacokinetic profiles of natural and synthetic compounds with anti-diabetic activity. Biomolecules. (2021) 11(11):1692. doi: 10.3390/biom11111692 34827690 PMC8615418

[207] Wang Y Shi LL Wang LY Xu JW Feng Y. Protective effects of MDG-1, a polysaccharide from ophiopogon japonicus on diabetic nephropathy in diabetic KKAy mice. Int J Mol Sci. (2015) 16:22473–84. doi: 10.3390/ijms160922473 PMC4613319 26393572

[208] Swallah MS Bondzie-Quaye P Wu Y Acheampong A Sossah FL Elsherbiny SM. Therapeutic potential and nutritional significance of Ganoderma lucidum - a comprehensive review from 2010 to 2022. Food Funct. (2023) 14(4):1812–38. doi: 10.1039/D2FO01683D 36734035

[209] Pan Y Zhang Y Li J Zhang Z He Y Zhao Q. A proteoglycan isolated from Ganoderma lucidum attenuates diabetic kidney disease by inhibiting oxidative stress-induced renal fibrosis both in vitro and in *vivo* . J Ethnopharmacol. (2023) 310:116405. doi: 10.1016/j.jep.2023.116405 36966849

[210] Tang X Huang M Jiang J Liang X Li X Meng R. Panax notoginseng preparations as adjuvant therapy for diabetic kidney disease: a systematic review and meta-analysis. Pharm Biol. (2020) 58(1):138–45. doi: 10.1080/13880209.2020.1711782 PMC7006712 31967924

[211] Zhang B Zhang X Zhang C Shen Q Sun G Sun X. Notoginsenoside R1 Protects db/db Mice against Diabetic Nephropathy via Upregulation of Nrf2-Mediated HO-1 Expression. Molecules. (2019) 24:247. doi: 10.3390/molecules24020247 30634720 PMC6359411

[212] Liang D Mai H Ruan F Fu H. The efficacy of triptolide in preventing diabetic kidney diseases: A systematic review and meta-analysis. Front Pharmacol. (2021) 12:728758. doi: 10.3389/fphar.2021.728758 34658869 PMC8517526

[213] Dong XG An ZM Guo Y Zhou JL Qin T. Effect of triptolide on expression of oxidative carbonyl protein in renal cortex of rats with diabetic nephropathy. J Huazhong Univ Sci Technolog Med Sci. (2017) 37:25–9. doi: 10.1007/s11596-017-1689-9 28224432

[214] Gao Q Shen W Qin W Zheng C Zhang M Zeng C. Treatment of db/db diabetic mice with triptolide: a novel therapy for diabetic nephropathy. Nephrol Dial Transplant. (2010) 25:3539–47. doi: 10.1093/ndt/gfq245 20483955

[215] Boers HM van Dijk TH Duchateau GS Mela DJ Hiemstra H Hoogenraad AR. Effect of mulberry fruit extract on glucose fluxes after a wheat porridge meal: a dual isotope study in healthy human subjects. Eur J Clin Nutr. (2023) 77(7):741–7. doi: 10.1038/s41430-023-01282-y 36944719

[216] Taghizadeh M Soleimani A Bahmani F Moravveji A Asadi A Amirani E. Metabolic response to mulberry extract supplementation in patients with diabetic nephropathy: a randomized controlled trial. Iran J Kidney Dis. (2017) 11:438–46. 29190604

[217] Jin D Zhang Y Zhang Y Duan L Zhou R Duan Y. Panax ginseng C.A.Mey. as medicine: the potential use of panax ginseng C.A.Mey. as a remedy for kidney protection from a pharmacological perspective. Front Pharmacol. (2021) 12:734151. doi: 10.3389/fphar.2021.734151 34512359 PMC8426624

[218] Wang T Huang X Zhai K Yu J Li J Duan H. Integrating metabolomics and network pharmacology to investigate Panax japonicus prevents kidney injury in HFD/STZ-induced diabetic mice. J Ethnopharmacol. (2023) 303:115893. doi: 10.1016/j.jep.2022.115893 36368565

[219] Salami M Salami R Mafi A Aarabi MH Vakili O Asemi Z. Therapeutic potential of resveratrol in diabetic nephropathy according to molecular signaling. Curr Mol Pharmacol. (2022) 15:716–35. doi: 10.2174/1874467215666211217122523 34923951

[220] Gu W Wang X Zhao H Geng J Li X Zheng K. Resveratrol ameliorates diabetic kidney injury by reducing lipotoxicity and modulates expression of components of the junctional adhesion molecule-like/sirtuin 1 lipid metabolism pathway. Eur J Pharmacol. (2022) 918:174776. doi: 10.1016/j.ejphar.2022.174776 35090936

[221] Liu W Gao Y Zhou Y Yu F Li X Zhang N. Mechanism of Cordyceps sinensis and its Extracts in the Treatment of Diabetic Kidney Disease: A Review. Front Pharmacol. (2022) 13:881835. doi: 10.3389/fphar.2022.881835 35645822 PMC9136174

[222] Yang J Dong H Wang Y Jiang Y Zhang W Lu Y. Cordyceps cicadae polysaccharides ameliorated renal interstitial fibrosis in diabetic nephropathy rats by repressing inflammation and modulating gut microbiota dysbiosis. Int J Biol Macromol. (2020) 163:442–56. doi: 10.1016/j.ijbiomac.2020.06.153 32592781

[223] Wojcikowski K Johnson DW Gobé G. Medicinal herbal extracts–renal friend or foe? Part two: herbal extracts with potential renal benefits. Nephrol (Carlton). (2004) 9:400–5. doi: 10.1111/j.1440-1797.2004.00355.x 15663644

[224] Zhao J Zhang QL Shen JH Wang K Liu J. Magnesium lithospermate B improves the gut microbiome and bile acid metabolic profiles in a mouse model of diabetic nephropathy. Acta Pharmacol Sin. (2019) 40:507–13. doi: 10.1038/s41401-018-0029-3 PMC6461985 29941869

[225] Dutta K Kravtsov V Oleynikova K Ruzov A Skorb EV Shityakov S. Analyzing the effects of single nucleotide polymorphisms on hnRNPA2/B1 protein stability and function: insights for anticancer therapeutic design. ACS Omega. (2024) 9:5485–95. doi: 10.1021/acsomega.3c07195 PMC10851241 38343990

[226] Fareed MM Dutta K Dandekar T Tarabonda H Skorb EV Shityakov S. In silico investigation of nonsynonymous single nucleotide polymorphisms in BCL2 apoptosis regulator gene to design novel protein-based drugs against cancer. J Cell Biochem. (2022) 123:2044–56. doi: 10.1002/jcb.30330 36146908

[227] Karnati S Guntas G Rajendran R Shityakov S Höring M Liebisch G. Quantitative lipidomic analysis of takotsubo syndrome patients’ Serum. Front Cardiovasc Med. (2022) 9:797154. doi: 10.3389/fcvm.2022.797154 35514439 PMC9062978

[228] Fareed MM Khalid H Khalid S Shityakov S. Deciphering molecular mechanisms of carbon tetrachlorideInduced hepatotoxicity (Fibrosis): A brief systematic review. Curr Mol Med. (2023). doi: 10.2174/0115665240257603230919103539 37818557

[229] Mugnai ML Shi Y Keatinge-Clay AT Elber R. Molecular dynamics studies of modular polyketide synthase ketoreductase stereospecificity. Biochemistry. (2015) 54:2346–59. doi: 10.1021/bi501401g PMC4501267 25835227

[230] Zhao J He K Du H Wei G Wen Y Wang J. Bioinformatics prediction and experimental verification of key biomarkers for diabetic kidney disease based on transcriptome sequencing in mice. PeerJ. (2022) 10:e13932. doi: 10.7717/peerj.13932 36157062 PMC9504448

